# Equilibrium investment with random risk aversion

**DOI:** 10.1111/mafi.12394

**Published:** 2023-05-03

**Authors:** Sascha Desmettre, Mogens Steffensen

**Affiliations:** ^1^ Institute for Financial Mathematics and Applied Number Theory University of Linz Linz Austria; ^2^ Department of Mathematical Sciences University of Copenhagen Copenhagen Denmark

**Keywords:** certainty equivalents, equilibrium approach, power and exponential utility, random risk aversion, time‐inconsistency

## Abstract

We solve the problem of an investor who maximizes utility but faces random preferences. We propose a problem formulation based on expected certainty equivalents. We tackle the time‐consistency issues arising from that formulation by applying the equilibrium theory approach. To this end, we provide the proper definitions and prove a rigorous verification theorem. We complete the calculations for the cases of power and exponential utility. For power utility, we illustrate in a numerical example that the equilibrium stock proportion is independent of wealth, but decreasing in time, which we also supplement by a theoretical discussion. For exponential utility, the usual constant absolute risk aversion is replaced by its expectation.

## 1 INTRODUCTION

We formulate and solve a dynamic portfolio optimization problem under random preferences by taking a distribution over certainty equivalents. This forms a new approach to optimization under random preferences. The problem becomes time‐inconsistent and we deal with this via the equilibrium approach. The elegance of the solution is demonstrated for standard choices of preferences like power (constant relative risk aversion) and exponential (constant absolute risk aversion) utility. For power utility, we show in a numerical example, how presence of random preferences supports the idea of declining life‐cycle investment profiles.

Portfolio optimization under random preferences is a topic of high relevance since an investor simply does not know the concrete value of her risk aversion. As a consequence of that, there is a large strand of literature that deals with the estimation of the this concrete value of risk aversion, for instance by using market data on labor income, compare Chetty (2006), market data of implied and realized volatilities, compare Bollerslev et al. (2011), or by investigating questionnaires of representative groups, compare Burgaard and Steffensen (2020).

The optimization problem under random preferences can be formalized in different ways: A first approach is to take expected utility where expectation is taken with respect to both financial risk and preference randomness or, equivalently, to take the expectation over risk aversions of the standard value function conditional on risk aversion. However, conditional on preferences, utility has only relative, no nominal, meaning in the sense that it allows for ordering of different strategies but the value function in itself is not directly comparable with other value functions based on other preferences. So, is an expectation (weighted sum) of such value functions conditional on preferences economically meaningful when each element in the sum has only relative meaning? We propose here instead to take the expectation over risk aversions of the so‐called certainty equivalents conditional on risk aversion. Since the unit of the certainty equivalent for each outcome of the utility function is monetary, this (weighted sum) makes economic sense.

What our approach provides in economic sense, it pays for in mathematical complexity. When forming the distribution over certainty equivalents, we form a sum over nonlinear functions of expectations, known to lead to issues of time‐inconsistency. The dynamic programming principle breaks down. Instead we approach the problem by the equilibrium approach. Under this approach, the current equilibrium decision is made subject to future decisions being equilibrium decisions themselves. In that way, we form a time‐consistent strategy but do not, however, find a classical optimum.

The idea of an equilibrium strategy dates back to Strotz (1956) whose sophisticated investor actually follows the equilibrium strategy. A precise mathematical formalization in continuous time was given by Ekeland and Lazrak (2006), Ekeland and Pirvu (2008), and Ekeland et al. (2012) with nonexponential discounting as the main example. The mean‐variance problem, first approached in this context by Basak and Chabakauri (2010), was put formally into the equilibrium context by Björk and Murgoci (2014), Björk et al. (2014), and Björk et al. (2017). Kryger et al. (2020) give a compendium style overview of different objectives with time‐inconsistency arising from the square function.

In general, in addition to the mentioned seminal papers above, time‐inconsistent control theory (c.f. e.g., Cui et al., 2017; He and Jiang, 2021; Hu et al., 2017) as well as time‐inconsistent optimal stopping problems (cf. e.g. Christensen and Lindensjö, 2018; Huang & Wang, 2021; Huang & Yu, 2021; Huang & Zhou, 2020) have notably gained attention in the (finance) community, introducing various new techniques. To this end, a comprehensive and profound overview on time‐inconsistent control theory with applications to finance can be found in Björk et al. (2021), including the references therein.

Finally, the papers that come closest to the idea in this paper introduce time‐inconsistency by aggregation of certainty equivalents. For instance, Jensen and Steffensen (2015) and Fahrenwaldt et al. (2020) aggregate certainty equivalents as a means to disentangle time and risk preferences. Before that Kryger and Steffensen (2010) had first introduced the idea of aggregating certainty equivalents in their titular problem of a “collective objective.” This is similar to our presentation here as the distribution of certainty equivalents over (an integer number of) individuals in their paper is similar to our distribution of certainty equivalents over different outcomes of the risk aversion of a single individual.

If equilibrium theory is our hammer, the random preferences are our nail. This is only scarcely studied in the financial literature. The basic idea given in Kräussl et al. (2012) comes close to our idea of dealing with financial risk and preference risk in two separate operations. However, they solve a static problem and, therefore, do not run into time‐inconsistency issues. That also goes for and Balter and Schweizer (2021) who even work with the idea of certainty equivalents like we do. Before that, random preferences showed up mainly in resource economics, see Li and Mattsson (1995), Van Kooten et al. (2001), and Akter et al. (2018). There, strikingly different, the preferences are the only source of randomness and working with certainty equivalents is redundant.

Another area related to our work is that of ambiguity aversion as introduced by Klibanoff et al. (2005, 2009) but studied by many others since then. The fundamental idea of separating risk into different dimensions and treating these dimensions differently is there used to treat uncertainty about market parameters different than *normal* market risk. The main difference to our work is that we treat randomness about preference parameters instead of uncertain market parameters. The motivation for these two distinct directions are quite different and, although on a fundamental level, the patterns of thinking are the same, our setup is not a special case of theirs. In that area, Balter et al. (2021) share common features with our work as their approach is as well based on certainty equivalents.

To understand and illustrate the relation to other works and ideas, we discuss thoroughly a few other areas of portfolio optimization. Particular emphasis is on relations to state‐dependent utility, see for example, Bernard et al. (2018), and robust optimization, see for example, Föllmer et al. (2009). Further references are given in a section devoted to that discussion. We question the economic meaning of addition or comparison of value functions corresponding to different utility functions. This has important impact on how state‐dependent utility should be understood and treated and it supports that our work is not covered by the state‐of‐art robust optimization.

The outline of the paper is as follows. In Section 2, we present the financial model and the optimization problem, and we argue the certainty equivalents and the equilibrium approach are the appropriate routes to take here. In Section 3, we derive a verification result for general utility functions and their related distributions. In Section 4, we present the special results for power and exponential utility, respectively, and we derive and illustrate strategies for the simplest nontrivial probability distribution of relative and absolute risk aversion, respectively. In Section 5, we discuss alternative related ideas and perform a numerical comparison of different strategies where the risk aversion is unknown.

## 2 THE FINANCIAL MARKET MODEL AND THE OPTIMIZATION PROBLEM

In this section, we present our approach to optimization under a random risk aversion. Let $\left(\Omega ,F,{\left(F_{t}\right)}_{0\leq t\leq T},P\right)$ be a filtered probability space, satisfying the usual conditions, and T>0 is a fixed time horizon. We consider a classical Black–Scholes financial market with price processes,

$$\begin{array}{cc} dS^{0}\left(t\right) & =S^{0}\left(t\right)rdt,\\ dS(t) & =S\left(t\right)\left(\alpha dt+\sigma dB(t)\right), \end{array}$$

where *B* is an $\left(F_{t}\right)$‐Brownian motion, α>r and r,α,σ>0 constants. We denote by θ:=(α−r)/σ the market price of risk.

In what follows we denote by π(t)∈R the *fraction of wealth* invested in the stock at time *t*, and the process π is called the *portfolio strategy*. The *wealth process* under an admissible portfolio strategy π is given by the solution of the stochastic differential equation

(1) $$\begin{array}{ccc} dX^{\pi }\left(t\right) & = & X^{\pi }\left(t\right)\left(r+\pi \left(t\right)\left(\alpha -r\right)\right)dt+X^{\pi }\left(t\right)\pi \left(t\right)\sigma dB\left(t\right), \end{array}$$

where $X\left(0\right)=x_{0}>0$ is the given initial wealth.

The *classical optimization problem* is to find the self‐financing investment strategy that maximizes the expected utility from terminal wealth, that is,

(2) $$\begin{array}{ccc} & & V\left(t,x\right):=\underset{\pi }{\sup}E_{t,x}\left[u\left(X^{\pi }\left(T\right)\right)\right], \end{array}$$

where $E_{t,x}$ is the conditional expectation given $X_{t}=x$, the supremum is taken over all admissible portfolio strategies, and u:(0,∞)→R is a utility function. A portfolio strategy is *optimal* if it attains the supremum. Equivalently, the investor's goal is to *maximize the certainty equivalent of terminal wealth* in an equilibrium sense, that is, to maximize the reward functional

(3) $$J^{\pi }\left(t,x\right):=u^{-1}\left(E_{t,x}\left[u\left(X^{\pi }\left(T\right)\right)\right]\right)\;.$$

where the strategy π is an equilibrium strategy in the sense of Definition 3.3. We detail this later but state it already here to prepare the reader for our approach which is a generalization of Equation (3) rather than of Equation (2). Note that while Equation (2) is measured on a utility scale, Equation (3) is measured in monetary scale.

In this paper, we model the parameter of a utility function γ as a real‐valued random variable. Examples are the constant (known) relative and absolute risk aversions, respectively, that are replaced by random variables. We form an optimization problem based on the idea to *maximize the certainty equivalent of terminal wealth w.r.t. a random risk aversion* in an equilibrium sense, that is, we want to maximize the reward functional

(4) $$J^{\pi }\left(t,x\right):=\int {\left(u^{\gamma }\right)}^{-1}\left(E_{t,x}\left[u^{\gamma }\left(X^{\pi }\left(T\right)\right)\right]\right)\;d\Gamma \left(\gamma \right),$$

where Γ is the cumulative distribution function (CDF) of γ, and we integrate over the support of the corresponding CDF. Moreover, we assume that the dependence of the utility function *u*. on γ∼Γ is such that the integral in Equation (4) is always well‐defined. Note that now we decorate the utility function by subscript γ to highlight its dependence on the risk aversion.

Remark 2.1 Perhaps, one might think of instead considering the objective

(5) $$V\left(t,x\right):=\underset{\pi }{\sup}\;E_{t,x}\left[\int u^{\gamma }\left(X^{\pi }\left(T\right)\right)\;d\Gamma \left(\gamma \right)\right],$$

that is, a plain expectation of utility with respect to both financial and preference risk. We cannot say that Equation (5) is outright wrong but, as also discussed briefly in the introduction, we can point at some deficiencies of that approach, all of which our approach (5) repair. First, the integral w.r.t. Γ essentially averages out different utility functions but why should utility be additive in that dimension? For example, different utility functions have different currency units and, therefore, a plain addition seems not meaningful. Second, whereas the value function in Equation (4) actually is the certainty equivalent, it is hard even to form a meaningful certainty equivalent from the value function (5). Third, but this is a mathematical deficiency rather than an economic deficiency, Equation (5) is surprisingly difficult to solve since the usual separation does not work, and wealth‐independence of the optimal portfolio for power utility is lost.

The objective in Equation (4) unveils a clear resemblance with the area of ambiguity aversion. As also introduced by Klibanoff et al. (2005, 2009), Equation (4) contains an *inner* expectation with respect to regular market risk. Their *outer* expectation is taken with respect to market parameter uncertainty whereas our *outer* expectation is with respect to preference parameter randomness. In that sense, one is not a special case of the other although they rely on the same fundamental pattern of thinking. A few further comments in that direction are in place. Klibanoff et al. (2005, 2009) take a general function ϕ between the two expectations whereas we take the special function ${\left(u^{\gamma }\right)}^{-1}$. At first glance, our choice looks like a special case of theirs. However, note that our choice makes the function a stochastic function that depends explicitly on the preference parameter γ whereas, in ambiguity aversion, the market parameter is not a part of the function ϕ. So, also in that direction, there are distinct differences. Our choice, of course, relates closely to the motivating Remark 2.1, which is not part of the ambiguity aversion motivation since the currency unit of expected utility is the same for all realized market parameters. Finally, the case where the distribution of market parameters is unknown has caught a lot of attention under the name robust optimization. We focus here on the case where the distribution of the preference parameter is known and relate only briefly to the idea of robustness with respect to preference parameter uncertainty in Section 5.

## 3 EMBEDDING THE PROBLEM IN TIME‐INCONSISTENT CONTROL THEORY

We now first formalize the equilibrium problem and then characterize its solution in a verification theorem. We introduce

(6) $$\begin{array}{c} y^{\pi ,\gamma }\left(t,x\right):=E_{t,x}\left[u^{\gamma }\left(X^{\pi }\left(T\right)\right)\right], \end{array}$$

such that the objective of the investor is to maximize the reward functional

(7) $$\begin{array}{c} J^{\pi }\left(t,x\right):=\int {\left(u^{\gamma }\right)}^{-1}\left(y^{\pi ,\gamma }\left(t,x\right)\right)d\Gamma \left(\gamma \right) \end{array}$$

in a given sense. We need admissible trading strategies in the equilibrium control framework. These are defined such that the value function of the objective exists.

Definition 3.1 (Admissible control law) An admissible control law is a map π:[0,T]×R→R satisfying the following conditions:

(a) For each initial point (t,x)∈[0,T]×R, the SDE (1) has a unique strong solution denoted by $X^{\pi }$.
(b) For each initial point (t,x)∈[0,T]×R, we have
(8) $$\begin{array}{c} \int {\left(u^{\gamma }\right)}^{-1}\left(E_{t,x}\left[u^{\gamma }\left(X^{\pi }\left(T\right)\right)\right]\right)d\Gamma \left(\gamma \right)<\infty \;. \end{array}$$
(c) π is continuous.

The set of admissible strategies is denoted by U.

Remark 3.2 The above definition is a straightforward adaptation of Def. 15.1 in Björk et al. (2021) to our setting, enhanced by the assumption of continuity in the spirit of Assumption 2.1 in Lindensjö (2019). As noted in Lindensjö (2019), controls used in most applications trivially satisfy this condition and it will also be met by our equilibrium control(s).

We seek to determine equilibrium control laws in the sense of the following definition.

Definition 3.3 (Equilibrium control law, cf. Def. 15.3 in Björk et al. (2021)) Consider an admissible control law $\hat{\pi }$ (informally viewed as a candidate equilibrium law). Choose an arbitrary π∈U and a fixed real number h>0. Fix moreover an arbitrarily chosen initial point (t,x) and define the control law $\pi_{h}$ by

$$\begin{array}{c} \pi_{h}\left(s,y\right)=\left\{\begin{array}{cc} \pi & \text{for}\;\;(s,y)\in [t,t+h)\times R,\\ \hat{\pi } & \text{for}\;\;(s,y)\in [t+h,T)\times R\;. \end{array}\right. \end{array}$$

$$\begin{array}{c} \text{If}\;\;\underset{h\to 0}{\lim\inf}\;\frac{J^{\hat{\pi }}\left(t,x\right)-J^{\pi_{h}}\left(t,x\right)}{h}\geq 0, \end{array}$$

for all π∈U, then $\hat{\pi }$ is referred to as an equilibrium control law.

Given that $\hat{\pi }$ is an equilibrium control law, we define the *equilibrium value function* *V* by

$$V\left(t,x\right):=J^{\hat{\pi }}\left(t,x\right)\;.$$

For the ease of notation, from now on, we use the following definition:

$$\begin{array}{c} \iota^{\gamma }\left(y\right):=\frac{d}{dy}{\left(u^{\gamma }\right)}^{-1}\left(y\right)\;. \end{array}$$

We are now ready to form and prove the verification theorem as it looks for the equilibrium value function and the equilibrium control for an investor who faces a random risk aversion. The structure of the verification theorem is similar to the structure of a verification theorem in classical optimal control theory. If one can find a sufficiently regular function specifically related to a local point‐wise optimum, then the function and the optimum forms an equilibrium value function and control, respectively (sufficiency). We do not know upfront whether an equilibrium value function and control, respectively, have the characteristics explained in the verification theorem (necessity), and we do not know upfront anything about uniqueness. Conversely, uniqueness of and necessary conditions for equilibrium controls have only recently gained attention in relation to linear‐quadratic problems by Hu et al. (2017) and in relation to optimal stopping problems by Huang and Zhou (2020) and Huang and Wang (2021). Uniqueness and necessary condition are beyond the scope of our exposition and we, consistently, just speak of *an* equilibrium strategy rather than *the* equilibrium strategy.

Moreover, in order to properly formulate our verification theorem, we need to introduce the following function space:

Definition 3.4 (*L* ^2^‐space, cf. Def. 15.5 in Björk et al. (2021) and Def. 5.1 in Björk et al. (2017)) Consider an arbitrary admissible control π∈U. A function $h:R_{+}\times R\to R$ is said to belong to the space $L^{2}\left(X^{\pi }\right)$ if it satisfies the condition

(9) $$\begin{array}{c} E_{t,x}\left[\int_{t}^{T}\left|\right|h_{x}\left(s,X^{\pi }\left(s\right)\right)\;\sigma \pi \left(s\right)X^{\pi }\left(s\right){\left|\right|}^{2}\;ds\right]<\infty , \end{array}$$

for every (t,x). In this expression, $h_{x}$ denotes the gradient of *h* in the *x*‐variable.

The proof of the verification theorem to follow is inspired by the proof of Theorem 15.1 in Björk et al. (2021), respectively, Theorem 5.2 in Björk et al. (2017).

Theorem 3.5 (Verification theorem) Assume that there exist functions $U\in C^{1,2}$, $Y^{\gamma }\in C^{1,2}$ for all γ, such that

(10) $$\begin{array}{cc} U_{t}\left(t,x\right)=\underset{\pi }{\inf}\{ & -\left(r+\pi \left(\alpha -r\right)\right)xU_{x}\left(t,x\right)-0.5\pi^{2}x^{2}\sigma^{2}U_{xx}\left(t,x\right)+H_{t}\left(t,x\right)\\ & +\left(r+\pi \left(\alpha -r\right)\right)xH_{x}\left(t,x\right)+0.5\pi^{2}x^{2}\sigma^{2}H_{xx}\left(t,x\right)\\ & -\int \iota^{\gamma }\left(Y^{\gamma }\left(t,x\right)\right)(Y_{t}^{\gamma }\left(t,x\right)\\ & +\left(r+\pi \left(\alpha -r\right)x\right)Y_{x}^{\gamma }\left(t,x\right)+0.5\sigma^{2}\pi^{2}x^{2}Y_{xx}^{\gamma }\left(t,x\right))\;d\Gamma \left(\gamma \right)\}, \end{array}$$

and

(11) $$\begin{array}{c} Y_{t}^{\gamma }\left(t,x\right)=-\left(r+\hat{\pi }\left(\alpha -r\right)\right)xY_{x}^{\gamma }\left(t,x\right)-0.5\sigma^{2}{\hat{\pi }}^{2}x^{2}Y_{xx}^{\gamma }\left(t,x\right), \end{array}$$

where $H\left(t,x\right)=\int {\left(u^{\gamma }\right)}^{-1}\left(Y^{\gamma }\left(t,x\right)\right)d\Gamma \left(\gamma \right)\in C^{1,2}$ and

(12) $$\begin{array}{cc} \hat{\pi }=\underset{\pi }{\arg\inf}\{ & -\left(r+\pi \left(\alpha -r\right)\right)xU_{x}\left(t,x\right)-0.5\pi^{2}x^{2}\sigma^{2}U_{xx}\left(t,x\right)+H_{t}\left(t,x\right)\\ & +\left(r+\pi \left(\alpha -r\right)\right)xH_{x}\left(t,x\right)+0.5\pi^{2}x^{2}\sigma^{2}H_{xx}\left(t,x\right)\\ & -\int \iota^{\gamma }\left(Y^{\gamma }\left(t,x\right)\right)(Y_{t}^{\gamma }\left(t,x\right)\\ & +\left(r+\pi \left(\alpha -r\right)x\right)Y_{x}^{\gamma }\left(t,x\right)+0.5\sigma^{2}\pi^{2}x^{2}Y_{xx}^{\gamma }\left(t,x\right))\;d\Gamma \left(\gamma \right)\}, \end{array}$$

with boundary conditions

(13) $$\begin{array}{c} U\left(T,x\right)=x,\;\text{and}\;\;\;Y^{\gamma }\left(T,x\right)=u^{\gamma }\left(x\right)\;\text{for all}\;\gamma \;. \end{array}$$

Furthermore assume that U,H, and $Y^{\gamma }$ for all γ, belong to the space $L^{2}\left(X^{\hat{\pi }}\right)$. Moreover, assume that the drift and diffusion coefficients of $X^{\hat{\pi }}$ satisfy linear growth conditions (see e.g., Karatzas and Shreve, 1991, (2.13), Section 5.2) and that the solution of Equation (11) with the boundary condition given in Equation (13) has polynomial growth (see e.g., Karatzas and Shreve, 1991, (7.14), Section 5.7).

Then $\hat{\pi }$ is an equilibrium control, and we have that

(14) $$\begin{array}{cc} V(t,x) & =U(t,x), \end{array}$$

(15) $$\begin{array}{cc} y^{\hat{\pi },\gamma }\left(t,x\right) & =Y^{\gamma }\left(t,x\right)\;\text{for all}\;\gamma \;. \end{array}$$

The proof consists of four steps.

- In the first step, we show that the probabilistic representation,
$$y^{\hat{\pi },\gamma }\left(t,x\right)=Y^{\gamma }\left(t,x\right)\;\text{for all}\;\gamma ,$$
  holds.
- In the second step, we show that
$$U\left(t,x\right)=J^{\hat{\pi }}\left(t,x\right)\;.$$
- In the third step, we show that $\hat{\pi }$ is indeed an equilibrium control law.
- In the fourth step, we establish V=U.

**Step 1**: As by assumption for all γ, $Y^{\gamma }\in C^{1,2}$ as well as $U,H\in C^{1,2}$, we obtain continuity of $\hat{\pi }$, and as a consequence, also continuity of the drift and diffusion coefficients of $X^{\hat{\pi }}$. Moreover, by the assumptions of the theorem, the drift and diffusion coefficients of $X^{\hat{\pi }}$ have linear growth, and the solution of Equation (11) with the boundary condition given in Equation (13) has polynomial growth. Therefore, together with $Y^{\gamma }\in L^{2}$ for all γ, an application of the Feynman–Kac theorem yields:

(16) $$\begin{array}{c} Y^{\gamma }\left(t,x\right)=E_{t,x}\left[u^{\gamma }\left(X^{\hat{\pi }}\left(T\right)\right)\right]=y^{\hat{\pi },\gamma }\left(t,x\right)\;\text{for all}\;\gamma \;. \end{array}$$

**Step 2**: The pseudo HJB (10) for $\pi =\hat{\pi }$ reads

$$\begin{array}{cc} U_{t}\left(t,x\right)= & -\left(r+\hat{\pi }\left(\alpha -r\right)\right)xU_{x}\left(t,x\right)-0.5{\hat{\pi }}^{2}x^{2}\sigma^{2}U_{xx}\left(t,x\right)+H_{t}\left(t,x\right)\\ & +\left(r+\hat{\pi }\left(\alpha -r\right)\right)xH_{x}+0.5{\hat{\pi }}^{2}x^{2}\sigma^{2}H_{xx}\left(t,x\right)\\ & -\int \iota^{\gamma }\left(Y^{\gamma }\left(t,x\right)\right)\left(Y_{t}^{\gamma }\left(t,x\right)+\left(r+\hat{\pi }\left(\alpha -r\right)\right)xY_{x}^{\gamma }\left(t,x\right)+0.5\sigma^{2}{\hat{\pi }}^{2}x^{2}Y_{xx}^{\gamma }\left(t,x\right)\right)\;d\Gamma \left(\gamma \right)\;. \end{array}$$

Plugging in Equation (11) then yields

(17) $$\begin{array}{cc} U_{t}\left(t,x\right)= & -\left(r+\hat{\pi }\left(\alpha -r\right)\right)xU_{x}\left(t,x\right)-0.5{\hat{\pi }}^{2}x^{2}\sigma^{2}U_{xx}\left(t,x\right)\\ & +H_{t}\left(t,x\right)+\left(r+\hat{\pi }\left(\alpha -r\right)\right)xH_{x}\left(t,x\right)+0.5{\hat{\pi }}^{2}x^{2}\sigma^{2}H_{xx}\left(t,x\right)\;. \end{array}$$

We obtain by Ito's lemma, using that $U\in L^{2}\left(X^{\hat{\pi }}\right)$:

(18) $$\begin{array}{cc} E_{t,x}\left[U\left(T,X^{\hat{\pi }}\left(T\right)\right)\right]=U\left(t,x\right)+E_{t,x}[\int_{t}^{T}(U_{s}\left(s,X^{\hat{\pi }}\left(s\right)\right) & +\left(r+\hat{\pi }\left(\alpha -r\right)\right)X^{\hat{\pi }}\left(s\right)U_{x}\left(s,X^{\hat{\pi }}\left(s\right)\right)\\ & +0.5\sigma^{2}{\hat{\pi }}^{2}{\left(X^{\hat{\pi }}\left(s\right)\right)}^{2}U_{xx}\left(s,X^{\hat{\pi }}\left(s\right)\right))ds]\;. \end{array}$$

Analogously, we obtain with $H\in L^{2}\left(X^{\hat{\pi }}\right)$:

(19) $$\begin{array}{cc} E_{t,x}\left[H\left(T,X^{\hat{\pi }}\left(T\right)\right)\right]=H\left(t,x\right)+E_{t,x}[\int_{t}^{T}(H_{s}\left(s,X^{\hat{\pi }}\left(s\right)\right) & +\left(r+\hat{\pi }\left(\alpha -r\right)\right)X^{\hat{\pi }}\left(s\right)H_{x}\left(s,X^{\hat{\pi }}\left(s\right)\right)\\ & +0.5\sigma^{2}{\hat{\pi }}^{2}{\left(X^{\hat{\pi }}\left(s\right)\right)}^{2}H_{xx}\left(s,X^{\hat{\pi }}\left(s\right)\right))ds]\;. \end{array}$$

Note that Equations (18) and (19) hold in general for $U,H\in L^{2}\left(X^{\hat{\pi }}\right)$ and rely on neither Equation (10) nor Equation (11). Since U(T,x)=H(T,x), we get from subtracting Equation (19) from Equation (18) and using Equation (17) for the first equality and Equation (16) for the third equality:

(20) $$\begin{array}{c} U\left(t,x\right)=H\left(t,x\right)=\int {\left(u^{\gamma }\right)}^{-1}\left(Y^{\gamma }\left(t,x\right)\right)d\Gamma \left(\gamma \right)=\int {\left(u^{\gamma }\right)}^{-1}\left(y^{\hat{\pi },\gamma }\left(t,x\right)\right)d\Gamma \left(\gamma \right)=J^{\hat{\pi }}\left(t,x\right)\;. \end{array}$$

**Step 3**: By Lemma 3.8 of Björk and Murgoci (2014), applied to the points *t* and t+h, we now obtain

$$\begin{array}{cc} J^{\pi_{h}}\left(t,x\right)=E_{t,x}\left[J^{\pi_{h}}\left(t+h,X^{\pi_{h}}\left(t+h\right)\right)\right] & -E_{t,x}\left[\int {\left(u^{\gamma }\right)}^{-1}\left(y^{\pi_{h},\gamma }\left(t+h,X^{\pi_{h}}\left(t+h\right)\right)\right)\;d\Gamma \left(\gamma \right)\right]\\ & +\int {\left(u^{\gamma }\right)}^{-1}\left(E_{t,x}\left[y^{\pi_{h},\gamma }\left(t+h,X^{\pi_{h}}\left(t+h\right)\right)\right]\right)\;d\Gamma \left(\gamma \right)\;. \end{array}$$

Since $\pi_{h}=\pi$ on [t,t+h), we have by continuity of $X^{\pi_{h}}\left(s\right)$, s≤t, that

$$X^{\pi_{h}}\left(t+h\right)=X^{\pi }\left(t+h\right),$$

and since $\pi_{h}=\hat{\pi }$ on [t+h,T], we get by using Equation (20)

$$J^{\pi_{h}}\left(t+h,X^{\pi_{h}}\left(t+h\right)\right)=U\left(t+h,X^{\pi }\left(t+h\right)\right),$$

and, furthermore,

$$y^{\pi_{h},\gamma }\left(t+h,X^{\pi_{h}}\left(t+h\right)\right)=y^{\hat{\pi },\gamma }\left(t+h,X^{\pi }\left(t+h\right)\right)\;.$$

Thus, we obtain

(21) $$\begin{array}{cc} J^{\pi_{h}}\left(t,x\right)=E_{t,x}\left[U\left(t+h,X^{\pi }\left(t+h\right)\right)\right] & -E_{t,x}\left[\int {\left(u^{\gamma }\right)}^{-1}\left(y^{\hat{\pi },\gamma }(t+h,X^{\pi }\left(t+h\right)\right)\;d\Gamma \left(\gamma \right)\right]\\ & +\int {\left(u^{\gamma }\right)}^{-1}\left(E_{t,x}\left[y^{\hat{\pi },\gamma }\left(t+h,X^{\pi }\left(t+h\right)\right)\right]\right)\;d\Gamma \left(\gamma \right)\;. \end{array}$$

We moreover obtain from the pseudo HJB (10) with π=π(t,x) and using Equation (16):

(22) $$\begin{array}{cc} U_{t}\left(t,x\right) & \leq -\left(r+\pi \left(\alpha -r\right)x\right)U_{x}\left(t,x\right)-0.5\sigma^{2}\pi^{2}x^{2}U_{xx}\left(t,x\right)+H_{t}\left(t,x\right)+\left(r+\pi \left(\alpha -r\right)\right)xH_{x}\left(t,x\right)\\ & +0.5\sigma^{2}\pi^{2}x^{2}H_{xx}\left(t,x\right)\\ & -\int \iota^{\gamma }\left(y^{\hat{\pi },\gamma }\left(t,x\right)\right)\left(y_{t}^{\hat{\pi },\gamma }\left(t,x\right)+\left(r+\pi \left(\alpha -r\right)\right)xy_{x}^{\hat{\pi },\gamma }\left(t,x\right)+0.5\sigma^{2}\pi^{2}x^{2}y_{xx}^{\hat{\pi },\gamma }\left(t,x\right)\right)\;d\Gamma \left(\gamma \right)\;. \end{array}$$

By first applying Ito's lemma for a fixed h>0 and then taking the limit h→0, noting that the admissible control π(t,x) is continuous, we obtain the following identities:

(23) $$\begin{array}{cc} E_{t,x} & \left[U(t+h,X^{\pi }\left(t+h\right))\right]-U\left(t,x\right)\\ & =h\{U_{t}\left(t,x\right)+\left(r+\pi \left(t,x\right)\left(\alpha -r\right)\right)xU_{x}\left(t,x\right)+0.5\sigma^{2}\pi^{2}\left(t,x\right)x^{2}U_{xx}\left(t,x\right)\}+o\left(h\right), \end{array}$$

(24) $$\begin{array}{cc} E_{t,x} & [H\left(t+h,X^{\pi }\left(t+h\right)\right)]-H\left(t,x\right)\\ & =h\{H_{t}\left(t,x\right)+\left(r+\pi \left(t,x\right)\left(\alpha -r\right)\right)xH_{x}\left(t,x\right)+0.5\sigma^{2}\pi^{2}\left(t,x\right)x^{2}H_{xx}\left(t,x\right)\}+o\left(h\right), \end{array}$$

(25) $$\begin{array}{cc} E_{t,x} & \left[y^{\hat{\pi },\gamma }\left(t+h,X^{\pi }\left(t+h\right)\right)\right]-y^{\hat{\pi },\gamma }\left(t,x\right)\\ & =h\{y_{t}^{\hat{\pi },\gamma }\left(t,x\right)+\left(r+\pi \left(t,x\right)\left(\alpha -r\right)\right)xy_{x}^{\hat{\pi },\gamma }\left(t,x\right)+0.5\sigma^{2}\pi^{2}\left(t,x\right)x^{2}y_{xx}^{\hat{\pi },\gamma }\left(t,x\right)\}+o\left(h\right)\;. \end{array}$$

From Equation (25), we moreover obtain

(26) $$\begin{array}{cc} & \int {\left(u^{\gamma }\right)}^{-1}\left(E_{t,x}\left[y^{\hat{\pi },\gamma }\left(t+h,X^{\pi }\left(t+h\right)\right)\right]\right)\;d\Gamma \left(\gamma \right)-\int {\left(u^{\gamma }\right)}^{-1}\left(y^{\hat{\pi },\gamma }\left(t,x\right)\right)d\Gamma \left(\gamma \right)\\ & =h\{\int \iota^{\gamma }\left(y^{\hat{\pi },\gamma }\left(t,x\right)\right)(y_{t}^{\hat{\pi },\gamma }\left(t,x\right)\\ & +\left(r+\pi \left(t,x\right)\left(\alpha -r\right)\right)xy_{x}^{\hat{\pi },\gamma }\left(t,x\right)+0.5\sigma^{2}\pi^{2}\left(t,x\right)x^{2}y_{xx}^{\hat{\pi },\gamma }\left(t,x\right))\;d\Gamma \left(\gamma \right)\}+o\left(h\right)\;. \end{array}$$

Then, combining Equations (23), (24), (26) with Equation (22), we thus get

$$\begin{array}{cc} & E_{t,x}\left[U\left(t+h,X^{\pi }\left(t+h\right)\right)\right]-U\left(t,x\right)-E_{t,x}\left[H\left(t+h,X^{\pi }\left(t+h\right)\right)\right]+H\left(t,x\right)\\ & +\int {\left(u^{\gamma }\right)}^{-1}\left(E_{t,x}\left[y^{\hat{\pi },\gamma }\left(t+h,X^{\pi }\left(t+h\right)\right)\right]\right)\;d\Gamma \left(\gamma \right)-\int {\left(u^{\gamma }\right)}^{-1}\left(y^{\hat{\pi },\gamma }\left(t,x\right)\right)\;d\Gamma \left(\gamma \right)\leq o\left(h\right), \end{array}$$

which by using $H\left(t,x\right)=\int {\left(u^{\gamma }\right)}^{-1}\left(Y^{\gamma }\left(t,x\right)\right)d\Gamma \left(\gamma \right)$ simplifies to

U(t,x)≥=Et,x[U(t+h,Xπ(t+h)]−Et,x[H(t+h,Xπ(t+h))]+∫(uγ)−1(Et,x[yπ^,γ(t+h,Xπ(t+h)])dΓ(γ)+o(h)=Jπh(t,x)+o(h).

Note that for the last equality, we have used Equation (21) and, furthermore, Equation (20) to establish

$$E_{t,x}\left[H\left(t+h,X^{\pi }\left(t+h\right)\right)\right]=E_{t,x}\left[\int {\left(u^{\gamma }\right)}^{-1}\left(y^{\hat{\pi },\gamma }(t+h,X^{\pi }\left(t+h\right)\right)\;d\Gamma \left(\gamma \right)\right]\;.$$

Now note that by Equation (20), we have $U\left(t,x\right)=J^{\hat{\pi }}\left(t,x\right)$, such that

$$J^{\hat{\pi }}\left(t,x\right)-J^{\pi_{h}}\left(t,x\right)\geq o\left(h\right),$$

and thus

$$\underset{h\to 0}{\lim\inf}\;\frac{J^{\hat{\pi }}\left(t,x\right)-J^{\pi_{h}}\left(t,x\right)}{h}\geq 0,$$

that is, $\hat{\pi }$ is an equilibrium control.

**Step 4**: With $\hat{\pi }$ being an equilibrium control, we conclude that U=V, which finalizes the proof.□

For the special form of *H* given by

$$H\left(t,x\right)=\int {\left(u^{\gamma }\right)}^{-1}\left(Y^{\gamma }\left(t,x\right)\right)\;d\Gamma \left(\gamma \right)$$

we obtain as an immediate consequence:

Corollary 3.6 From the pseudo HJB (10), we obtain by using

$$\begin{array}{cc} H(t,x) & =\int {\left(u^{\gamma }\right)}^{-1}\left(Y^{\gamma }\left(t,x\right)\right)\;d\Gamma \left(\gamma \right),\\ H_{t}\left(t,x\right) & =\int \iota^{\gamma }\left(Y^{\gamma }\left(t,x\right)\right)Y_{t}^{\gamma }\left(t,x\right)\;d\Gamma \left(\gamma \right),\\ H_{x}\left(t,x\right) & =\int \iota^{\gamma }\left(Y^{\gamma }\left(t,x\right)\right)Y_{x}^{\gamma }\left(t,x\right)\;d\Gamma \left(\gamma \right),\\ H_{xx}\left(t,x\right) & =\int \iota^{\gamma }\left(Y^{\gamma }\left(t,x\right)\right)Y_{xx}^{\gamma }\left(t,x\right)\;d\Gamma \left(\gamma \right)+\int {\left(\iota^{\gamma }\right)}^{\prime }\left(Y^{\gamma }\left(t,x\right)\right){\left(Y_{x}^{\gamma }\left(t,x\right)\right)}^{2}\;d\Gamma \left(\gamma \right), \end{array}$$

the following form:

(27) $$\begin{array}{cc} U_{t}\left(t,x\right)=\underset{\pi }{\inf}\{ & -\left(r+\pi \left(\alpha -r\right)\right)xU_{x}\left(t,x\right)-0.5\sigma^{2}\pi^{2}x^{2}U_{xx}\left(t,x\right)\\ & +0.5\sigma^{2}\pi^{2}x^{2}\int {\left(\iota^{\gamma }\right)}^{\prime }\left(Y^{\gamma }\left(t,x\right)\right){\left(Y_{x}^{\gamma }\left(t,x\right)\right)}^{2}\;d\Gamma \left(\gamma \right)\}\;. \end{array}$$

In this formulation, the nonlinearity arising within the time‐inconsistent control problem is clearly visible, compare Björk et al. (2021, Section 16.2).

## 4 SOLVING THE OPTIMIZATION PROBLEM

In this section, we solve our problem for two different utility functions, namely the classical power utility and exponential utility functions, respectively. Before specializing to the first of the two, however, we solve the optimization problem for a general utility function as far as possible. The arginf over π of Equation (27) is the solution to the equation,

(28) $$\begin{array}{cc} & -\left(\alpha -r\right)xU_{x}\left(t,x\right)-\sigma^{2}\pi x^{2}\left(U_{xx}\left(t,x\right)-\int {\left(\iota^{\gamma }\right)}^{\prime }\left(Y^{\gamma }\left(t,x\right)\right){\left(Y_{x}^{\gamma }\left(t,x\right)\right)}^{2}\;d\Gamma \left(\gamma \right)\right)=0, \end{array}$$

which gives the following candidate for an equilibrium strategy:

(29) $$\begin{array}{ccc} \hat{\pi }\left(t,\Gamma \right) & = & -\frac{\alpha -r}{\sigma^{2}}\frac{1}{x}\frac{U_{x}\left(t,x\right)}{U_{xx}\left(t,x\right)-\int {\left(\iota^{\gamma }\right)}^{\prime }\left(Y^{\gamma }\left(t,x\right)\right){\left(Y_{x}^{\gamma }\left(t,x\right)\right)}^{2}\;d\Gamma \left(\gamma \right)}\;. \end{array}$$

With that at place, the task for a specific utility function is to come up with candidate functions *U* and $Y^{\gamma }$, for all γ.

By imposing

(30) $$\begin{array}{c} U\left(t,x\right)=\int {\left(u^{\gamma }\right)}^{-1}\left(Y^{\gamma }\left(t,x\right)\right)\;d\Gamma \left(\gamma \right), \end{array}$$

such that

(31) $$\begin{array}{cc} U_{x}\left(t,x\right) & =\int \iota^{\gamma }\left(Y^{\gamma }\left(t,x\right)\right)Y_{x}^{\gamma }\left(t,x\right)\;d\Gamma \left(\gamma \right), \end{array}$$

(32) $$\begin{array}{cc} U_{xx}\left(t,x\right) & =\int \iota^{\gamma }\left(Y^{\gamma }\left(t,x\right)\right)Y_{xx}\left(t,x\right)\;d\Gamma \left(\gamma \right)+\int {\left(\iota^{\gamma }\right)}^{\prime }\left(Y^{\gamma }\left(t,x\right)\right){\left(Y_{x}^{\gamma }\left(t,x\right)\right)}^{2}\;d\Gamma \left(\gamma \right), \end{array}$$

we eliminate the dependency on *U* in Equation (29), and arrive at

(33) $$\begin{array}{ccc} \hat{\pi }\left(t,\Gamma \right) & = & -\frac{\alpha -r}{\sigma^{2}}\frac{1}{x}\frac{\int \iota^{\gamma }\left(Y^{\gamma }\left(t,x\right)\right)Y_{x}^{\gamma }\left(t,x\right)\;d\Gamma \left(\gamma \right)}{\int \iota^{\gamma }\left(Y^{\gamma }\left(t,x\right)\right)Y_{xx}^{\gamma }\left(t,x\right)\;d\Gamma \left(\gamma \right)}\;. \end{array}$$

Thus, from now on, we only have to guess on $Y^{\gamma }$, for all γ.

### 4.1 Power utility preferences

For a power utility function,

$$\begin{array}{c} u^{\gamma }\left(\cdot \right)=\frac{1}{1-\gamma }{\left(\cdot \right)}^{1-\gamma }, \end{array}$$

we obtain its inverse and the corresponding derivative as

$$\begin{array}{c} {\left(u^{\gamma }\right)}^{-1}\left(\cdot \right)={\left(1-\gamma \right)}^{\frac{1}{1-\gamma }}{\left(\cdot \right)}^{\frac{1}{1-\gamma }}\;,\;\frac{d}{dv}{\left(u^{\gamma }\right)}^{-1}\left(v\right)={\left(1-\gamma \right)}^{\frac{\gamma }{1-\gamma }}{\left(v\right)}^{\frac{\gamma }{1-\gamma }}\;. \end{array}$$

Thus, the candidate equilibrium strategy reads

$$\hat{\pi }\left(t,\Gamma \right)=-\frac{\alpha -r}{\sigma^{2}}\frac{1}{x}\frac{\int {\left(1-\gamma \right)}^{\frac{\gamma }{1-\gamma }}{\left(Y^{\gamma }\left(t,x\right)\right)}^{\frac{\gamma }{1-\gamma }}Y_{x}^{\gamma }\left(t,x\right)d\Gamma \left(\gamma \right)}{\int {\left(1-\gamma \right)}^{\frac{\gamma }{1-\gamma }}{\left(Y^{\gamma }\left(t,x\right)\right)}^{\frac{\gamma }{1-\gamma }}Y_{xx}^{\gamma }\left(t,x\right)d\Gamma \left(\gamma \right)}\;.$$

We guess the following ansatz with its partial derivatives:

(34) $$\begin{array}{cc} Y^{\gamma }\left(t,x\right) & =\frac{1}{1-\gamma }{\left(g^{\gamma }\left(t\right)\right)}^{\gamma }x^{1-\gamma }\;,\;Y_{t}^{\gamma }\left(t,x\right)=\frac{\gamma }{1-\gamma }{\left(g^{\gamma }\left(t\right)\right)}^{\gamma -1}g_{t}^{\gamma }\left(t\right)x^{1-\gamma },\\ Y_{x}^{\gamma }\left(t,x\right) & ={\left(g^{\gamma }\left(t\right)\right)}^{\gamma }x^{-\gamma }\;\;\;\;,\;Y_{xx}^{\gamma }\left(t,x\right)=-\gamma {\left(g^{\gamma }\left(t\right)\right)}^{\gamma }x^{-\gamma -1}\;. \end{array}$$

We emphasize again that we use top‐script γ on functions to denote their dependence on γ rather than taking the function to the power of γ.

Substituting this back into the candidate equilibrium strategy yields

$$\begin{array}{c} \hat{\pi }\left(t,\Gamma \right)=\frac{\alpha -r}{\sigma^{2}}\frac{1}{\varepsilon \left(t,\Gamma \right)},\;\;\text{with}\;\varepsilon \left(t,\Gamma \right)=\frac{\int {\left(g^{\gamma }\left(t\right)\right)}^{\frac{\gamma }{1-\gamma }}\gamma \;d\Gamma \left(\gamma \right)}{\int {\left(g^{\gamma }\left(t\right)\right)}^{\frac{\gamma }{1-\gamma }}\;d\Gamma \left(\gamma \right)}\;. \end{array}$$

We immediately see that, provided that our ansatz is correct, then the equilibrium strategy inherits the usual property of the power utility case, namely wealth‐independence of the equilibrium strategy, whereas time‐independence is lost. Also note that ε(t,Γ) is actually a weighted average of γs, where the weights are densities in the distribution Γ distorted in a particular way by the functions $g^{\gamma }$ .

We are now left with the task to check our ansatz by plugging it into the condition for admissibility, which is that means that, suppressing the argument (t,Γ) of $\hat{\pi }$,

$$\begin{array}{c} \frac{\partial }{\partial t}Y^{\gamma }\left(t,x\right)=-\left(r+\hat{\pi }\left(\alpha -r\right)\right)xY_{x}^{\gamma }\left(t,x\right)-\frac{1}{2}\sigma^{2}{\hat{\pi }}^{2}x^{2}Y_{xx}^{\gamma }\left(t,x\right)\;,\;\;Y^{\gamma }\left(T,x\right)=\frac{1}{1-\gamma }x^{1-\gamma }\;. \end{array}$$

Plugging in the ansatz and the corresponding derivatives gives the equation 

$$\begin{array}{ccc} \frac{\gamma }{1-\gamma }{\left(g^{\gamma }\left(t\right)\right)}^{\gamma -1}g_{t}^{\gamma }\left(t\right) & = & -\left(r+\hat{\pi }\left(\alpha -r\right)\right){\left(g^{\gamma }\left(t\right)\right)}^{\gamma }+0.5\sigma^{2}{\hat{\pi }}^{2}\gamma {\left(g^{\gamma }\left(t\right)\right)}^{\gamma }\;,\;\;g^{\gamma }\left(T\right)=1\;,\;\;\text{i.e.} \end{array}$$

(35) $$\begin{array}{ccc} g_{t}^{\gamma }\left(t\right) & = & \frac{1-\gamma }{\gamma }\left(-r+0.5\theta^{2}\frac{1}{{\left(\varepsilon \left(t,\Gamma \right)\right)}^{2}}\gamma -\theta^{2}\frac{1}{\varepsilon \left(t,\Gamma \right)}\right)g^{\gamma }\left(t\right)\;,\;\;\;g^{\gamma }\left(T\right)=1. \end{array}$$

We moreover obtain by differentiation of ε(t,Γ) w.r.t. time:

$$\begin{array}{ccc} \frac{\partial \varepsilon \left(t,\Gamma \right)}{\partial t} & = & \frac{\left(\int {\left(g^{\gamma }\left(t\right)\right)}^{\frac{\gamma }{1-\gamma }}d\Gamma \left(\gamma \right)\right)\left(\int \gamma \frac{\gamma }{1-\gamma }{\left(g^{\gamma }\left(t\right)\right)}^{\frac{\gamma }{1-\gamma }-1}g_{t}^{\gamma }\left(t\right)d\Gamma \left(\gamma \right)\right)}{{\left(\int {\left(g^{\gamma }\left(t\right)\right)}^{\frac{\gamma }{1-\gamma }}d\Gamma \left(\gamma \right)\right)}^{2}}\\ & & -\frac{\left(\int \frac{\gamma }{1-\gamma }{\left(g^{\gamma }\left(t\right)\right)}^{\frac{\gamma }{1-\gamma }-1}g_{t}^{\gamma }\left(t\right)d\Gamma \left(\gamma \right)\right)\left(\int {\left(g^{\gamma }\left(t\right)\right)}^{\frac{\gamma }{1-\gamma }}\gamma d\Gamma \left(\gamma \right)\right)}{{\left(\int {\left(g^{\gamma }\left(t\right)\right)}^{\frac{\gamma }{1-\gamma }}d\Gamma \left(\gamma \right)\right)}^{2}}\\ & = & \frac{\left(\int {\left(g^{\gamma }\left(t\right)\right)}^{\frac{\gamma }{1-\gamma }}d\Gamma \left(\gamma \right)\right)\left(\int \gamma {\left(g^{\gamma }\left(t\right)\right)}^{\frac{\gamma }{1-\gamma }}\left(-r+0.5\theta^{2}\frac{1}{\varepsilon^{2}\left(t,\Gamma \right)}\gamma -\theta^{2}\frac{1}{\varepsilon \left(t,\Gamma \right)}\right)d\Gamma \left(\gamma \right)\right)}{{\left(\int {\left(g^{\gamma }\left(t\right)\right)}^{\frac{\gamma }{1-\gamma }}d\Gamma \left(\gamma \right)\right)}^{2}}\\ & & -\frac{\left(\int {\left(g^{\gamma }\left(t\right)\right)}^{\frac{\gamma }{1-\gamma }}\left(-r+0.5\theta^{2}\frac{1}{\varepsilon^{2}\left(t,\Gamma \right)}\gamma -\theta^{2}\frac{1}{\varepsilon \left(t,\Gamma \right)}\right)d\Gamma \left(\gamma \right)\right)\left(\int {\left(g^{\gamma }\left(t\right)\right)}^{\frac{\gamma }{1-\gamma }}\gamma d\Gamma \left(\gamma \right)\right)}{{\left(\int {\left(g^{\gamma }\left(t\right)\right)}^{\frac{\gamma }{1-\gamma }}d\Gamma \left(\gamma \right)\right)}^{2}}\;. \end{array}$$

Using Equation (35), this simplifies to

(36) $$\begin{array}{ccc} \varepsilon_{t}\left(t,\Gamma \right)=\frac{\partial \varepsilon \left(t,\Gamma \right)}{\partial t} & = & 0.5\theta^{2}\frac{1}{\varepsilon^{2}\left(t,\Gamma \right)}(\frac{\left(\int {\left(g^{\gamma }\left(t\right)\right)}^{\frac{\gamma }{1-\gamma }}d\Gamma \left(\gamma \right)\right)\left(\int \gamma^{2}{\left(g^{\gamma }\left(t\right)\right)}^{\frac{\gamma }{1-\gamma }}d\Gamma \left(\gamma \right)\right)}{{\left(\int {\left(g^{\gamma }\left(t\right)\right)}^{\frac{\gamma }{1-\gamma }}d\Gamma \left(\gamma \right)\right)}^{2}}\\ & & -\frac{\left(\int {\left(g^{\gamma }\left(t\right)\right)}^{\frac{\gamma }{1-\gamma }}\gamma d\Gamma \left(\gamma \right)\right)\left(\int {\left(g^{\gamma }\left(t\right)\right)}^{\frac{\gamma }{1-\gamma }}\gamma d\Gamma \left(\gamma \right)\right)}{{\left(\int {\left(g^{\gamma }\left(t\right)\right)}^{\frac{\gamma }{1-\gamma }}d\Gamma \left(\gamma \right)\right)}^{2}})\\ & = & 0.5\theta^{2}\frac{1}{\varepsilon^{2}\left(t,\Gamma \right)}\left(\frac{\int \gamma^{2}{\left(g^{\gamma }\left(t\right)\right)}^{\frac{\gamma }{1-\gamma }}d\Gamma \left(\gamma \right)}{\int {\left(g^{\gamma }\left(t\right)\right)}^{\frac{\gamma }{1-\gamma }}d\Gamma \left(\gamma \right)}-\varepsilon^{2}\left(t,\Gamma \right)\right)\;. \end{array}$$

From the infinite‐dimensional ODE system (35) and (36), it is now clear that our ansatz is correct such that $\hat{\pi }\left(t,\Gamma \right)$ is an equilibrium control, given that the ODE system admits a solution and that the admissibility conditions as well as the assumptions made in the verification theorem are satisfied. As these are very challenging tasks on a general level, we will provide the corresponding arguments for the special cases of a two‐point and a one‐point distribution for γ below.

The differential equation (35) is linear such that it actually allows an exponential solution expressed in terms of the function ε. We can now plug this function into ε(t,T) and its derivative. We have

$$\begin{array}{cc} g^{\gamma }\left(t\right) & =\exp\left(\int_{t}^{T}\frac{1-\gamma }{\gamma }\left(-r+0.5\theta^{2}\frac{1}{{\left(\varepsilon \left(s,\Gamma \right)\right)}^{2}}\gamma -\theta^{2}\frac{1}{\varepsilon \left(s,\Gamma \right)}\right)ds\right), \end{array}$$

such that

(37) $$\begin{array}{c} \varepsilon \left(t,\Gamma \right)=\frac{\int \left(\exp\left(\int_{t}^{T}\left(-r+0.5\theta^{2}\frac{1}{{\left(\varepsilon \left(s,\Gamma \right)\right)}^{2}}\gamma -\theta^{2}\frac{1}{\varepsilon \left(s,\Gamma \right)}\right)ds\right)\right)\gamma d\Gamma \left(\gamma \right)}{\int \left(\exp\left(\int_{t}^{T}\left(-r+0.5\theta^{2}\frac{1}{{\left(\varepsilon \left(s,\Gamma \right)\right)}^{2}}\gamma -\theta^{2}\frac{1}{\varepsilon \left(s,\Gamma \right)}\right)ds\right)\right)d\Gamma \left(\gamma \right)}\;. \end{array}$$

Similarly, we can plug the expression for *g* into Equation (35) to achieve

(38) $$\begin{array}{c} \varepsilon_{t}\left(t,\Gamma \right)=0.5\theta^{2}\frac{1}{\varepsilon^{2}\left(t,\Gamma \right)}\left(\frac{\int \gamma^{2}\exp\left(\int_{t}^{T}\left(-r+0.5\theta^{2}\frac{1}{{\left(\varepsilon \left(s,\Gamma \right)\right)}^{2}}\gamma -\theta^{2}\frac{1}{\varepsilon \left(s,\Gamma \right)}\right)ds\right)d\Gamma \left(\gamma \right)}{\int \exp\left(\int_{t}^{T}\left(-r+0.5\theta^{2}\frac{1}{{\left(\varepsilon \left(s,\Gamma \right)\right)}^{2}}\gamma -\theta^{2}\frac{1}{\varepsilon \left(s,\Gamma \right)}\right)ds\right)d\Gamma \left(\gamma \right)}-\varepsilon^{2}\left(t,\Gamma \right)\right).\; \end{array}$$

The expressions for ε and $\varepsilon_{t}$ in Equations (37) and (38) show how the linear structure of the ODE for *g*, Equation (35), allows us to reduce the infinite‐dimensional system of ODEs to a single equation description. However, that equation is a highly implicit representation of ε. One may hope for making it more explicit for certain distributions Γ, but an easy route for that is not immediately visible. The simplest idea is to discretize the distribution Γ. For a discrete distribution Γ, the outer integral with respect to Γ becomes a sum and the system of ODEs becomes more tractable. If the sum is finite, the system of ODEs becomes finite and we have existence of a solution as we argue for in the numerical study below.

#### Special case with two possible risk aversions

If the distribution of the risk aversion is a two‐point distribution with possible outcomes γ_1_ and γ_2_ that are, realized with the probabilities $p_{1}=p\left(\gamma_{1}\right)$ and $p_{2}=p\left(\gamma_{2}\right)$, we obtain

(39) $$\begin{array}{c} \varepsilon \left(t,\Gamma \right)=\frac{p_{1}{\left(g^{\gamma_{1}}\left(t\right)\right)}^{\frac{\gamma_{1}}{1-\gamma_{1}}}\gamma_{1}+p_{2}{\left(g^{\gamma_{2}}\left(t\right)\right)}^{\frac{\gamma_{2}}{1-\gamma_{2}}}\gamma_{2}}{p_{1}{\left(g^{\gamma_{1}}\left(t\right)\right)}^{\frac{\gamma_{1}}{1-\gamma_{1}}}+p_{2}{\left(g^{\gamma_{2}}\left(t\right)\right)}^{\frac{\gamma_{2}}{1-\gamma_{2}}}}, \end{array}$$

and the two inter‐related ordinary differential equations,

(40) $$\begin{array}{ccc} g_{t}^{\gamma_{1}}\left(t\right) & = & \frac{1-\gamma_{1}}{\gamma_{1}}\left(-r+0.5\theta^{2}\frac{1}{{\left(\varepsilon \left(t,\Gamma \right)\right)}^{2}}\gamma_{1}-\theta^{2}\frac{1}{\varepsilon \left(t,\Gamma \right)}\right)g^{\gamma_{1}}\left(t\right)\;,\;\;g^{\gamma_{1}}\left(T\right)=1, \end{array}$$

(41) $$\begin{array}{ccc} g_{t}^{\gamma_{2}}\left(t\right) & = & \frac{1-\gamma_{2}}{\gamma_{2}}\left(-r+0.5\theta^{2}\frac{1}{{\left(\varepsilon \left(t,\Gamma \right)\right)}^{2}}\gamma_{2}-\theta^{2}\frac{1}{\varepsilon \left(t,\Gamma \right)}\right)g^{\gamma_{2}}\left(t\right)\;,\;\;g^{\gamma_{2}}\left(T\right)=1\;. \end{array}$$

Now, using again the fact that the risk aversion follows a two‐point distribution with possible outcomes γ_1_ and γ_2_ that are, realized with the probabilities $p_{1}=p\left(\gamma_{1}\right)$ and $p_{2}=p\left(\gamma_{2}\right)$, we obtain the ordinary differential equation 

(42) $$\begin{array}{ccc} \varepsilon_{t}\left(t,\Gamma \right)=\frac{\partial \varepsilon \left(t,\Gamma \right)}{\partial t} & = & 0.5\theta^{2}\frac{1}{\varepsilon^{2}\left(t,\Gamma \right)}\cdot \delta \left(t,\Gamma \right)-0.5\theta^{2}, \end{array}$$

where

(43) $$\begin{array}{c} \delta \left(t,\Gamma \right)=\frac{p_{1}{\left(g^{\gamma_{1}}\left(t\right)\right)}^{\frac{\gamma_{1}}{1-\gamma_{1}}}\gamma_{1}^{2}+p_{2}{\left(g^{\gamma_{2}}\left(t\right)\right)}^{\frac{\gamma_{2}}{1-\gamma_{2}}}\gamma_{2}^{2}}{p_{1}{\left(g^{\gamma_{1}}\left(t\right)\right)}^{\frac{\gamma_{1}}{1-\gamma_{1}}}+p_{2}{\left(g^{\gamma_{2}}\left(t\right)\right)}^{\frac{\gamma_{2}}{1-\gamma_{2}}}}, \end{array}$$

and where ε(t,Γ) is given by Equation (39). Equations (40)–(42), thus, form a three‐dimensional system of ordinary differential equations, instead of an infinite‐dimensional one in the general power utility case. Therefore, the question of an existence of a solution in general infinite‐dimensional ODE system boils down to the solvability of this three‐dimensional system. Clearly, the right‐hand sides of Equations (40)–(42) are continuous on their respective domain, such that by the existence theorem of Peano at least a (possibly nonunique) local solution exists (possibly up to some explosion time $T_{\infty }$). In the concrete case, Equation (39) imposes even some more structure on the solution of Equations (40) and (41). We note in particular that the solutions of Equations (40) and (41) can only explode when ε(t,Γ)—which is just an expected value of the two possible values γ_1_ and γ_2_—reaches zero. However, in the two‐point case $\varepsilon \left(t,\Gamma \right)\in \left[\gamma_{1},\gamma_{2}\right]$, and therefore, the value zero cannot be attained.^1^ We wish to stress that in accordance with working in an equilibrium control framework and thus having a (possibly nonunique) equilibrium control, the existence of a local solution is sufficient for our purposes.

The argument for existence of a solution presented in the previous paragraph extends trivially to discrete distributions with bounded support. In this case the infinite‐dimensional system of ODEs is actually finite and ε is bounded from below and from above by the maximum and minimum outcome of γ. For general distributions, the simplest idea is to study an approximate problem, discretized and with bounded support.

#### Special case with one possible risk aversion

If the distribution of the risk aversion is degenerate with the only outcome γ, we have that ε(t,Γ)≡γ, such that the time derivative of *g* becomes the easily recognizable,

(44) $$\begin{array}{c} g_{t}^{\gamma }\left(t\right)=-\frac{1-\gamma }{\gamma }\left(r+0.5\theta^{2}\frac{1}{\gamma }\right)g^{\gamma }\left(t\right)\;,\;\;g^{\gamma }\left(T\right)=1\;. \end{array}$$

This means in particular that as expected, we recover the classical solution for the case of one possible risk aversion, as we do not have to deal with any randomness w.r.t. the risk aversion here. This confirms that the equilibrium strategy solving (3) coincides with the optimal strategy solving (2), that is the Merton strategy.

Remark 4.1 (Solvability of Equation (44)) Clearly, Equation (44) admits a (unique) closed‐form solution, that is

$$\begin{array}{c} g^{\gamma }\left(t\right)=e^{\frac{1-\gamma }{\gamma }\left(r+0.5\theta^{2}\frac{1}{\gamma }\right)\left(T-t\right)}\;. \end{array}$$

Therefore, the only task left for the special case of one possible risk aversion is to check the admissibility conditions and the assumptions made in the verification theorem, which we do in the following paragraph.

#### Checking of assumptions

As already lined out, in order to finally show that our ansatz gives the claimed equilibrium control(s), we have in addition to show that the found solutions satisfy the admissibility conditions and the assumptions made in the verification theorem.

We note that in the case of power utility, we obtain

(45) $$\begin{array}{c} Y^{\gamma }\left(t,x\right)=y^{\hat{\pi },\gamma }\left(t,x\right)=E_{t,x}\left[u^{\gamma }\left(X^{\hat{\pi }}\left(T\right)\right)\right]=\frac{1}{1-\gamma }{\left(g^{\gamma }\left(t\right)\right)}^{\gamma }x^{1-\gamma }\;\text{for all}\;\gamma \;. \end{array}$$

To see that $Y^{\gamma }\in C^{1,2}$, note that for the two‐point case in Equation (45), $g^{\gamma_{1}}$ and $g^{\gamma_{2}}$ have nonexploding solutions. For the one‐point case note that we have a closed‐form solution for $g^{\gamma }$.

Note, moreover, that for power utility, the condition that $Y^{\gamma }\in L^{2}\left(X^{\hat{\pi }}\right)$, boils down to

(46) $$\begin{array}{c} \sigma \hat{\pi }\left(t\right)X^{\hat{\pi }}\left(t\right)Y_{x}^{\gamma }\left(t,X^{\hat{\pi }}\left(t\right)\right)=\sigma \hat{\pi }\left(t\right){\left(X^{\hat{\pi }}\left(t\right)\right)}^{1-\gamma }\frac{\gamma }{1-\gamma }{\left(g^{\gamma }\left(t\right)\right)}^{\gamma }\;\text{for all}\;\gamma , \end{array}$$

being square‐integrable. The square integrability of Equation (46) for $\gamma_{1},\gamma_{2}$ (two‐point case) and a single γ (one‐point case) now follows from the facts that the resulting equilibrium strategies $\hat{\pi }$ are deterministic and that $X^{\hat{\pi }}$ is given as the solution to the linear SDE (1) with deterministic coefficients, using that we have nonexploding solutions for $g^{\gamma_{1}}$ and $g^{\gamma_{2}}$, respectively, a closed‐form solution for $g^{\gamma }$.

Concerning the regularity of the functions *U* and *H* note that by using

$${\left(u^{\gamma }\right)}^{-1}\left(\cdot \right)={\left(1-\gamma \right)}^{\frac{1}{1-\gamma }}{\left(\cdot \right)}^{\frac{1}{1-\gamma }}$$

we obtain

(47) $$\begin{array}{c} U\left(t,x\right)=\int {\left(u^{\gamma }\right)}^{-1}\left(Y^{\gamma }\left(t,x\right)\right)d\Gamma \left(\gamma \right)=x\int {\left(g^{\gamma }\left(t\right)\right)}^{\frac{\gamma }{1-\gamma }}d\Gamma \left(\gamma \right)\;. \end{array}$$

In the concrete case of a two‐point distribution, Equation (47) reads

(48) $$\begin{array}{c} x\left(p_{1}{\left(g^{\gamma_{1}}\left(t\right)\right)}^{\frac{\gamma_{1}}{1-\gamma_{1}}}+p_{2}{\left(g^{\gamma_{2}}\left(t\right)\right)}^{\frac{\gamma_{2}}{2-\gamma_{2}}}\right)\;. \end{array}$$

The *C* ^1, 2^‐property of Equation (48) is now immediate, stressing that in the case of a two‐point distribution, we have a nonexploding solution for the corresponding ODE system. Accordingly, in the case of a one‐point distribution, Equation (48) has to be satisfied for the single specific γ and reads

(49) $$\begin{array}{c} x{\left(g^{\gamma }\left(t\right)\right)}^{\frac{\gamma }{1-\gamma }}=xe^{\left(r+0.5\theta^{2}\frac{1}{\gamma }\right)\left(T-t\right)}\;. \end{array}$$

The *C* ^1, 2^‐property of Equation (49), that is, in the case of a one‐point distribution for γ, is obvious from the right‐hand side of the equation itself, due to having a unique global solution for $g^{\gamma }$.

Note that for power utility, the condition that $U\in L^{2}\left(X^{\hat{\pi }}\right)$, in the case of a two‐point distribution, boils down to

(50) $$\begin{array}{c} \sigma \hat{\pi }\left(t\right)X^{\hat{\pi }}\left(t\right)U_{x}\left(t,X^{\hat{\pi }}\left(t\right)\right)=\sigma \hat{\pi }\left(t\right)X^{\hat{\pi }}\left(t\right)\left(p_{1}{\left(g^{\gamma_{1}}\left(t\right)\right)}^{\frac{\gamma_{1}}{1-\gamma_{1}}}+p_{2}{\left(g^{\gamma_{2}}\left(t\right)\right)}^{\frac{\gamma_{2}}{2-\gamma_{2}}}\right), \end{array}$$

being square‐integrable. The square integrability of Equation (50) for $\gamma_{1},\gamma_{2}$ (two‐point case) and a single γ (one‐point case) now follows from arguments similar to the ones that were carried out for the square integrability of Equation (46).

The *C* ^1, 2^‐property as well as the $L^{2}\left(X^{\hat{\pi }}\right)$‐property of *H* follow from the results for *U* together with the fact that $H\left(t,x\right)=\int {\left(u^{\gamma }\right)}^{-1}\left(Y^{\gamma }\left(t,x\right)\right)d\Gamma \left(\gamma \right)$.

Since the obtained equilibrium controls $\hat{\pi }$ are independent of *x* and recalling the form (1) of the wealth process, the coefficients of $X^{\hat{\pi }}$ are obviously of linear growth. By the same argument, that is, the independence of the obtained equilibrium controls $\hat{\pi }$ of *x*, the solution of Equation (11) with the boundary condition given in Equation (13), which is of the form (34), has as well polynomial growth.

We are finally left with proving the admissibility conditions from Definition 3.1. For proving Equation (8), we have to show that Equation (47) is finite. Accordingly, for the two‐point case, Equation (48) has to be finite, which is obviously true, as the solution is nonexploding. Similar but simpler arguments hold for the one‐point case.

To see that part (a) of Definition 3.1 holds true, we rely on the same line of arguments used to prove the square integrability of $Y^{\gamma }$ and *U*.

Part (c) of Definition 3.1 is satisfied, as due to continuous coefficients, the solutions to the ODE system (40)–(42) (two‐point case) and the ODE (44) (one‐point case) and thus the resulting equilibrium controls are continuous.

#### Numerical illustration

**FIGURE 1:**
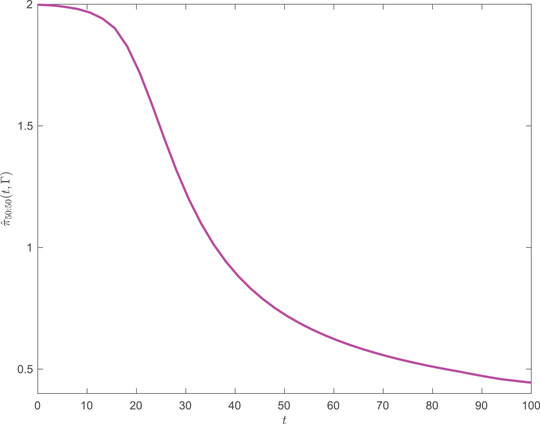
$t\to \hat{\pi }\left(t,\Gamma \right)$ with γ_1_, γ_2_ equally probable (50/50), that is, $p_{1}=p_{2}=0.5$. Market parameters: (r,α,σ)=(0.02,0.06,0.2). Risk aversions: $\gamma_{1}=0.5$, $\gamma_{2}=4$. Investment horizon: T=100. [Colour figure can be viewed at wileyonlinelibrary.com]

**FIGURE 2:**
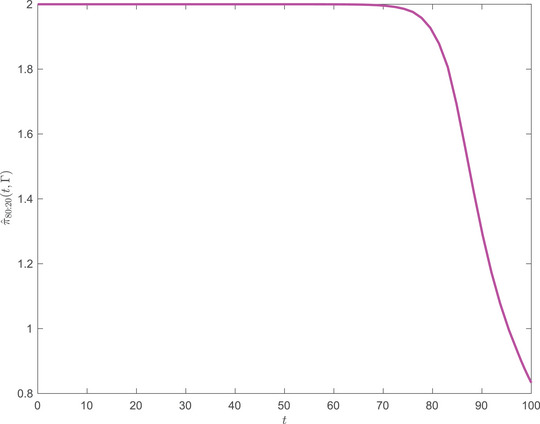
$t\to \hat{\pi }\left(t,\Gamma \right)$ with γ_1_ four times more probable than γ_2_ (80/20), that is, $p_{1}=0.8$ and $p_{2}=0.2$. Market parameters: (r,α,σ)=(0.02,0.06,0.2). Risk aversions: $\gamma_{1}=0.5$, $\gamma_{2}=4$. Investment horizon: T=100. [Colour figure can be viewed at wileyonlinelibrary.com]

In Figures 1 and 2, we display the equilibrium investment strategy for the two point‐distribution with parameters as specified in the caption. In both panels, the strategy starts close to the strategy as if the smallest risk aversion, 0.5, was certain ($\hat{\pi }=2$). In Figure 1, where the two risk aversions are equally likely, the strategy declines relatively slowly over 100 years to the strategy as if the expected risk version was certain (expected risk aversion 2.25 and $\hat{\pi }\approx 0.44$). In Figure 2, where the low risk aversion is four times as likely as the high risk aversion, the strategy stays close to its maximum, 2, until year 70 and, thereafter, declines relatively fast over 30 years, again towards the strategy as if the expected risk aversion was certain (expected risk aversion 1.2 and $\hat{\pi }\approx 0.83$).

The profiles of the strategies are interesting from a life‐cycle investment point of view. Standard marketed strategies are typically declining over age. There are various (other) good reasons for that, for example, the presence of a relatively risk‐free but decreasing value of future labor income. Our work suggests another argument, not instead but in addition. Actually, one of the practitioner's arguments against using Merton's portfolio theory is that it is practically difficult/impossible to infer a single individual's risk aversion. We have here coped with the randomness in risk aversion and showed that it produces in itself a demand for declining life‐cycle investment profiles.

Remark 4.2 The special case with only two possible risk aversions is related to the notion of α‐maxmin expected utility, see Ghirardato et al. (2004). This is an approach to ambiguity where a convex mixture of the best and worst case is formed. Best and worst here relate to different probability measures corresponding to different market parameters. Disregarding briefly the fundamental difference between uncertainty of market and preference parameters, respectively, as discussed in Section 2, our solution also forms a convex mixture of the strategies formed by the two outcomes of the risk aversion.

Although there are fundamental differences, also α‐maxmin expected utility leads to issues of time‐inconsistency. This has been approached by equilibrium theory in a optimal stopping problem by Huang and Yu (2021). Beissner et al. (2019) approach the same issue of time‐consistency by forming, recursively, a value function such that the strategy is locally constructed to be time‐consistent. The recursion is constructed in discrete time and, finally, a time limit is taken to form the continuous‐time version. The relation between a time‐consistent construction based on a local discrete‐time recursion and a time limit, and the equilibrium approach to a continuous‐time problem with time‐inconsistency is certainly worth studying but far beyond the scope of our work. That relation also appears in the distinction between the standard recursive utility as a way to separate time and risk preferences, and the equilibrium approach to the same separation studied by Jensen and Steffensen (2015) and Fahrenwaldt et al. (2020). Fahrenwaldt et al. (2020) show that in some—but just as important not all—markets, the two approaches to separation of time and risk preferences lead to the same strategies.

### 4.2 Exponential utility preferences

For an exponential utility function,

$$\begin{array}{c} u^{\gamma }\left(\cdot \right)=-\frac{1}{\gamma }e^{-\gamma \left(\cdot \right)}, \end{array}$$

we obtain its inverse and the corresponding derivative as

$$\begin{array}{c} {\left(u^{\gamma }\right)}^{-1}\left(\cdot \right)=-\frac{1}{\gamma }\log\left(-\gamma \left(\cdot \right)\right)\;,\;\frac{d}{dv}{\left(u^{\gamma }\right)}^{-1}\left(v\right)=-\frac{1}{\gamma v}\;. \end{array}$$

Thus the candidate equilibrium strategy is given by

$$\begin{array}{ccc} \hat{\pi }\left(t,\Gamma \right) & = & \frac{\left(\alpha -r\right)x\int \iota^{\gamma }\left(Y^{\gamma }\left(t,x\right)\right)Y_{x}^{\gamma }\left(t,x\right)d\Gamma \left(\gamma \right)}{-\sigma^{2}x^{2}\int \iota^{\gamma }\left(Y^{\gamma }\left(t,x\right)\right)Y_{xx}^{\gamma }\left(t,x\right)d\Gamma \left(\gamma \right)}=-\frac{\alpha -r}{\sigma^{2}}\frac{1}{x}\frac{\int \frac{1}{\gamma }\frac{Y_{x}^{\gamma }\left(t,x\right)}{Y^{\gamma }\left(t,x\right)}d\Gamma \left(\gamma \right)}{\int \frac{1}{\gamma }\frac{Y_{xx}^{\gamma }\left(t,x\right)}{Y^{\gamma }\left(t,x\right)}d\Gamma \left(\gamma \right)}\;. \end{array}$$

Here, our ansatz with its partial derivatives are

$$\begin{array}{ccc} Y^{\gamma }\left(t,x\right) & = & e^{g_{1}^{\gamma }\left(t\right)x+g_{2}^{\gamma }\left(t\right)},\;\;\;\;\;Y_{t}^{\gamma }\left(t,x\right)=e^{g_{1}^{\gamma }\left(t\right)x+g_{2}^{\gamma }\left(t\right)}\left(g_{1t}^{\gamma }\left(t\right)x+g_{2t}^{\gamma }\left(t\right)\right),\\ Y_{x}^{\gamma }\left(t,x\right) & = & g_{1}^{\gamma }\left(t\right)e^{g_{1}^{\gamma }\left(t\right)x+g_{2}^{\gamma }\left(t\right)},\;Y_{xx}^{\gamma }\left(t,x\right)={\left(g_{1}^{\gamma }\left(t\right)\right)}^{2}e^{g_{1}^{\gamma }\left(t\right)x+g_{2}^{\gamma }\left(t\right)}\;. \end{array}$$

Substituting this into the candidate equilibrium strategy gives

$$\begin{array}{ccc} \hat{\pi }\left(t,\Gamma \right)\;x & = & -\frac{\alpha -r}{\sigma^{2}}\frac{\int \frac{1}{\gamma }\frac{Y_{x}^{\gamma }\left(t,x\right)}{Y^{\gamma }\left(t,x\right)}d\Gamma \left(\gamma \right)}{\int \frac{1}{\gamma }\frac{Y_{xx}^{\gamma }\left(t,x\right)}{Y^{\gamma }\left(t,x\right)}d\Gamma \left(\gamma \right)}=-\frac{\alpha -r}{\sigma^{2}}\frac{\int \frac{1}{\gamma }g_{1}^{\gamma }\left(t\right)d\Gamma \left(\gamma \right)}{\int \frac{1}{\gamma }{\left(g_{1}^{\gamma }\left(t\right)\right)}^{2}d\Gamma \left(\gamma \right)}\;. \end{array}$$

As for the power utility case, we see that, provided that our ansatz is correct, the structure of the solution is inherited from the case without random preferences, namely that a wealth‐independent amount should be invested in the risky asset. We now need to check the ansatz by plugging it into the condition for admissibility, again suppressing the arguments (t,Γ) of $\hat{\pi }$:

$$\begin{array}{ccc} \frac{\partial }{\partial t}Y^{\gamma }\left(t,x\right) & = & -\left(r+\hat{\pi }\left(\alpha -r\right)\right)xY_{x}^{\gamma }\left(t,x\right)-\frac{1}{2}\sigma^{2}{\hat{\pi }}^{2}x^{2}Y_{xx}^{\gamma }\left(t,x\right)\;,\;\;Y^{\gamma }\left(T,x\right)=-\frac{1}{\gamma }e^{-\gamma x}\;. \end{array}$$

Plugging in the ansatz and its derivatives yields

$$\begin{array}{ccc} e^{g_{1}^{\gamma }\left(t\right)x+g_{2}^{\gamma }\left(t\right)}\left(\frac{\partial g_{1}^{\gamma }\left(t\right)}{\partial t}x+\frac{\partial g_{2}^{\gamma }\left(t\right)}{\partial t}\right) & = & -\left(r+\hat{\pi }\left(\alpha -r\right)\right)xg_{1}^{\gamma }\left(t\right)e^{g_{1}^{\gamma }\left(t\right)x+g_{2}^{\gamma }\left(t\right)}\\ & + & 0.5\sigma^{2}{\hat{\pi }}^{2}x^{2}{\left(g_{1}^{\gamma }\left(t\right)\right)}^{2}e^{g_{1}^{\gamma }\left(t\right)x+g_{2}^{\gamma }\left(t\right)},\\ \frac{\partial g_{1}^{\gamma }\left(t\right)}{\partial t}x+\frac{\partial g_{2}^{\gamma }\left(t\right)}{\partial t} & = & -\left(r+\hat{\pi }\left(\alpha -r\right)\right)xg_{1}^{\gamma }\left(t\right)+0.5\sigma^{2}{\hat{\pi }}^{2}x^{2}{\left(g_{1}^{\gamma }\left(t\right)\right)}^{2},\\ \frac{\partial g_{1}^{\gamma }\left(t\right)}{\partial t}x+\frac{\partial g_{2}^{\gamma }\left(t\right)}{\partial t} & = & -rxg_{1}^{\gamma }\left(t\right)+\theta^{2}\frac{\int \frac{1}{\gamma }g_{1}^{\gamma }\left(t\right)d\Gamma \left(\gamma \right)}{\int \frac{1}{\gamma }{\left(g_{1}^{\gamma }\left(t\right)\right)}^{2}d\Gamma \left(\gamma \right)}g_{1}^{\gamma }\left(t\right)\\ & + & 0.5\theta^{2}{\left(\frac{\int \frac{1}{\gamma }g_{1}^{\gamma }\left(t\right)d\Gamma \left(\gamma \right)}{\int \frac{1}{\gamma }{\left(g_{1}^{\gamma }\left(t\right)\right)}^{2}d\Gamma \left(\gamma \right)}\right)}^{2}{\left(g_{1}^{\gamma }\left(t\right)\right)}^{2}\;. \end{array}$$

Collecting terms that depend on wealth, we obtain

$$\begin{array}{ccc} \frac{\partial g_{1}^{\gamma }\left(t\right)}{\partial t} & = & -rg_{1}^{\gamma }\left(t\right)\;\;\text{with}\;\;g_{1}^{\gamma }\left(T\right)=-\gamma ,\;\;\text{i.e.,}\;\;g_{1}^{\gamma }\left(t\right)=-\gamma e^{r\left(T-t\right)}\;. \end{array}$$

With this insight, we have again confirmed that our ansatz is correct, and we know that the following strategy $\hat{\pi }$ is an equilibrium strategy:

$$\begin{array}{ccc} \hat{\pi }\left(t,\Gamma \right)\;x & = & -\frac{\alpha -r}{\sigma^{2}}\frac{1}{e^{r\left(T-t\right)}}\frac{-\int d\Gamma \left(\gamma \right)}{\int \frac{1}{\gamma }{\left(\gamma \right)}^{2}d\Gamma \left(\gamma \right)}=\frac{\alpha -r}{\sigma^{2}}\frac{1}{e^{r\left(T-t\right)}}\frac{\int d\Gamma \left(\gamma \right)}{\int \gamma d\Gamma \left(\gamma \right)}=\frac{\alpha -r}{\sigma^{2}}e^{-r\left(T-t\right)}\frac{1}{E\left[\gamma \right]}\;. \end{array}$$

We note for completeness that the ordinary differential equation characterizing $g_{2}^{\gamma }$ is a simple linear equation and that it is not an ingredient in the equilibrium strategy, in particular, we have that

$$\begin{array}{ccc} \frac{\partial g_{2}^{\gamma }\left(t\right)}{\partial t} & = & \theta^{2}\frac{\int \frac{1}{\gamma }g_{1}^{\gamma }\left(t\right)d\Gamma \left(\gamma \right)}{\int \frac{1}{\gamma }{\left(g_{1}^{\gamma }\left(t\right)\right)}^{2}d\Gamma \left(\gamma \right)}g_{1}^{\gamma }\left(t\right)+0.5\theta^{2}{\left(\frac{\int \frac{1}{\gamma }g_{1}^{\gamma }\left(t\right)d\Gamma \left(\gamma \right)}{\int \frac{1}{\gamma }{\left(g_{1}^{\gamma }\left(t\right)\right)}^{2}d\Gamma \left(\gamma \right)}\right)}^{2}{\left(g_{1}^{\gamma }\left(t\right)\right)}^{2}\\ & = & \frac{\gamma }{E(\gamma )}\left(\frac{\gamma \theta^{2}}{2}-\theta^{2}\right)\;. \end{array}$$

The equilibrium trading strategy is, thus, to hold a time‐dependent amount in the risky asset as in the case without random preferences. Actually, the strategy is the same as the classical one, where we simply replace the random γ by its expectation. This might be a first guess on a strategy, even before formalizing a problem appropriately. But we have here seen that this is actually correct for exponential utility, whereas it is incorrect for power utility.

Remark 4.3 We wish to stress that this result holds independently of the postulated distribution for γ. In particular, we are able to compute an equilibrium strategy $\hat{\pi }$ as long as the first moment of γ exists. Moreover, as the resulting ordinary differential equations for *g*_1_ and *g*_2_ have (continuous) closed‐form solutions, existence of a unique global solution is always guaranteed. Consequently, the argumentation concerning admissibility and the assumptions of the verification theorem now follows completely along the lines of the power utility case, but in a more direct way.

### 4.3 Dynamics of the equilibrium strategies

Obviously, the dynamics of the equilibrium strategies is only interesting for the case of power utility as for the case of exponential utility, we obtain

$$\frac{\partial \hat{\pi }\left(t,\Gamma \right)}{\partial t}=r\cdot \frac{\alpha -r}{\sigma^{2}x}e^{-r\left(T-t\right)}\frac{1}{E\left[\gamma \right]}=r\hat{\pi }\left(t,\Gamma \right)\;.$$

More interestingly, using the results of the previous section, for the case of power utility, we obtain by using Equation (36) that

$$\begin{array}{ccc} \frac{\partial \hat{\pi }\left(t,\Gamma \right)}{\partial t} & = & -\frac{\alpha -r}{\sigma^{2}}\frac{1}{\varepsilon^{2}\left(t,\Gamma \right)}\frac{\partial \varepsilon \left(t,\Gamma \right)}{\partial t}\\ & = & -\frac{\alpha -r}{\sigma^{2}}\frac{1}{{\left(\frac{\int {\left(g^{\gamma }\left(t\right)\right)}^{\frac{\gamma }{1-\gamma }}\gamma d\Gamma \left(\gamma \right)}{\int {\left(g^{\gamma }\left(t\right)\right)}^{\frac{\gamma }{1-\gamma }}d\Gamma \left(\gamma \right)}\right)}^{2}}\frac{\partial \varepsilon \left(t,\Gamma \right)}{\partial t}\\ & = & -\frac{\alpha -r}{\sigma^{2}}\frac{1}{{\left(\frac{\int {\left(g^{\gamma }\left(t\right)\right)}^{\frac{\gamma }{1-\gamma }}\gamma d\Gamma \left(\gamma \right)}{\int {\left(g^{\gamma }\left(t\right)\right)}^{\frac{\gamma }{1-\gamma }}d\Gamma \left(\gamma \right)}\right)}^{2}}0.5\theta^{2}\frac{1}{\varepsilon^{2}\left(t,\Gamma \right)}\left(\frac{\int \gamma^{2}{\left(g^{\gamma }\left(t\right)\right)}^{\frac{\gamma }{1-\gamma }}d\Gamma \left(\gamma \right)}{\int {\left(g^{\gamma }\left(t\right)\right)}^{\frac{\gamma }{1-\gamma }}d\Gamma \left(\gamma \right)}-\varepsilon^{2}\left(t,\Gamma \right)\right)\\ & = & -0.5\frac{{\left(\alpha -r\right)}^{3}}{\sigma^{4}}\left(\frac{\int \gamma^{2}{\left(g^{\gamma }\left(t\right)\right)}^{\frac{\gamma }{1-\gamma }}d\Gamma \left(\gamma \right)}{\int {\left(g^{\gamma }\left(t\right)\right)}^{\frac{\gamma }{1-\gamma }}d\Gamma \left(\gamma \right)}-{\left(\frac{\int {\left(g^{\gamma }\left(t\right)\right)}^{\frac{\gamma }{1-\gamma }}\gamma d\Gamma \left(\gamma \right)}{\int {\left(g^{\gamma }\left(t\right)\right)}^{\frac{\gamma }{1-\gamma }}d\Gamma \left(\gamma \right)}\right)}^{2}\right)\;. \end{array}$$

This implies that the equilibrium strategy $\hat{\pi }$ is actually decreasing, if we can show that

$$\frac{\int \gamma^{2}{\left(g^{\gamma }\left(t\right)\right)}^{\frac{\gamma }{1-\gamma }}d\Gamma \left(\gamma \right)}{\int {\left(g^{\gamma }\left(t\right)\right)}^{\frac{\gamma }{1-\gamma }}d\Gamma \left(\gamma \right)}-{\left(\frac{\int {\left(g^{\gamma }\left(t\right)\right)}^{\frac{\gamma }{1-\gamma }}\gamma d\Gamma \left(\gamma \right)}{\int {\left(g^{\gamma }\left(t\right)\right)}^{\frac{\gamma }{1-\gamma }}d\Gamma \left(\gamma \right)}\right)}^{2}\geq 0\;.$$

To see this, define the density^2^

$$\begin{array}{c} d\nu \left(\gamma \right):=\frac{{\left(g^{\gamma }\left(t\right)\right)}^{\frac{\gamma }{1-\gamma }}d\Gamma \left(\gamma \right)}{\int {\left(g^{\gamma }\left(t\right)\right)}^{\frac{\gamma }{1-\gamma }}d\Gamma \left(\gamma \right)}, \end{array}$$

and denote the expectation w.r.t. this density by $E^{\nu }\left[\cdot \right]$. Then it follows that

$$\begin{array}{c} \frac{\int \gamma^{2}{\left(g^{\gamma }\left(t\right)\right)}^{\frac{\gamma }{1-\gamma }}d\Gamma \left(\gamma \right)}{\int {\left(g^{\gamma }\left(t\right)\right)}^{\frac{\gamma }{1-\gamma }}d\Gamma \left(\gamma \right)}-{\left(\frac{\int {\left(g^{\gamma }\left(t\right)\right)}^{\frac{\gamma }{1-\gamma }}\gamma d\Gamma \left(\gamma \right)}{\int {\left(g^{\gamma }\left(t\right)\right)}^{\frac{\gamma }{1-\gamma }}d\Gamma \left(\gamma \right)}\right)}^{2}=E^{\nu }\left[\gamma^{2}\right]-{\left(E^{\nu }\left[\gamma \right]\right)}^{2}\geq 0. \end{array}$$

This theoretical result confirms the observed decreasing behavior of equilibrium strategies as shown in Figures 1 and 2.

## 5 RELATION TO OTHER APPROACHES AND OUTLOOK

In this section, we discuss related problems and ideas. The discussion leads to a few alternative ways to approach the randomness of risk aversion. At the end of the section, we compare how investors with some of these approaches perform within our numerical example of power utility.

### 5.1 Theoretical discussion of related approaches

Our problem is related to the problem of state‐dependent utility. That family of problems has been studied in the economics literature for decades. However, the typical situation is that the utility is dependent on the financial state and not driven by randomness orthogonal to that. This comes from the intuitive idea that rising stock prices make investors more optimistic and therefore, less risk‐averse and vice versa. Compared with the usual assumption of perfect correlation between the market states and the random utility, we go to the other extreme and assume no relation between the market states and the random utility. We stress, however, that our instigation to work with certainty equivalents, by referring to currency units, holds for state‐dependent utility in general. It is, namely, not the cause of variation in the risk aversion but the very fact that it varies, that calls for a thorough discussion on currency units. When working with certainty equivalents in the classical sense of (market‐) state‐dependent utility, the same currency issues, discussed in Section 2, appear again, and, again, the idea of comparing certainty equivalents repairs the economic problem. Carefully, however, we do not claim that other problems, economic or mathematically, cannot arise in that case. See Karni (1983) for an early discussion on state‐dependent utility which, actually, goes beyond the usual idea of just financial state‐dependence. See Londoño (2009) and Bernard et al. (2018) for more recent contributions where the state is primarily thought of as being financial.

One simple approach to the case of state‐dependent utility is to “approximate the problem” with a problem that is easily solved. A natural idea is to simply replace the random risk aversion by its expectation leading to a regular portfolio optimization problem based on the expected risk aversion. In the next subsection, we speak of such a strategy as the *expected risk aversion strategy*. In order to make the value function of that problem comparable with the equilibrium value function, we introduce a value function based on the certainty equivalent and calculate numerically

(51) $$V\left(t,x\right):=\underset{\pi }{\sup}\;{\left(u^{E\left[\gamma \right]}\right)}^{-1}\left(E_{t,x}\left[u^{E\left[\gamma \right]}\left(X^{\pi }\left(T\right)\right)\right]\right)\;.$$

The role of the inverse utility function here is just for the comparison purpose. It changes the value function but since the inverse utility is an increasing function, it does not change the optimal strategy, which is just the regular optimal portfolio based on expected risk aversion, compare the relation between Equations (2) and (3). The strategy attaining the supremum in Equation (51) is, generally, different from our equilibrium strategy. What we found in the previous section was that for the exponential utility optimizing investor, however, our strategy and the expected absolute risk aversion strategy coincide. For the power utility optimizing investor, our strategy and the expected relative risk aversion strategy do not coincide. We mention here that Balter and Schweizer (2021) actually find the expected relative risk aversion strategy to be optimal for a specific one‐period problem different from but related to ours. We acknowledge that Equation (51) as a general objective is not theoretically founded. However, in certain cases of exponential utility and a special case studied by Balter and Schweizer (2021), the resulting strategy is actually theoretically founded. Further, it seems like an immediate practical starting point without paying theoretical attention to the fundamental aspects.

A different strand of literature investigates robust optimization in order to seek robustness against the uncertainty that can be represented as variability in the parameters of the problem. These parameters were, however, to the knowledge of the authors always thought of as market model parameters and not preference parameters. If we include also preference parameters, one can also perform robust optimization with respect to the uncertainty of the risk aversion.

The idea of robust optimization has been present in Operations Research for half a century. Within the area of portfolio optimization, see for instance Schied (2005, 2007) and Föllmer et al. (2009) for original developments and Desmettre et al. (2015) for more recent contributions. A direct translation of robustness with respect to model parameters into robustness with respect to preference parameters leads to a value function in the form

(52) $$V\left(t,x\right):=\underset{\pi }{\sup}\underset{\gamma \in A}{\inf}\;E_{t,x}\left[u^{\gamma }\left(X^{\pi }\left(T\right)\right)\right],$$

where *A* is the set of risk aversions with respect to which robustness is sought for. Thus, the idea is to optimize over the investment strategy under the worst possible assumption about risk aversion within a given set.

Here, however, one has to be careful with the currency unit argument again. As long as uncertainty concerns market parameters in classic robust optimization, there are no currency unit issues. But when the uncertainty concerns preference parameters, the infimum over risk aversion actually implicitly compares quantities of different currency units. From an economic point of view, this is essentially as odd as adding them up when considering (5). Therefore, our proposal would be to consider instead

(53) $$V\left(t,x\right):=\underset{\pi }{\sup}\underset{\gamma \in A}{\inf}\;{\left(u^{\gamma }\right)}^{-1}\left(E_{t,x}\left[u^{\gamma }\left(X^{\pi }\left(T\right)\right)\right]\right)\;.$$

It is beyond the scope of this paper to study further the problem expressed through Equation (53). What we investigate instead in the next subsection for a special choice of *A*, is the strategy corresponding to the value function

(54) $$V\left(t,x\right):=\underset{\gamma \in A}{\inf}\underset{\pi }{\sup}\;{\left(u^{\gamma }\right)}^{-1}\left(E_{t,x}\left[u^{\gamma }\left(X^{\pi }\left(T\right)\right)\right]\right)\;.$$

We speak of the optimal strategy for the value function (54) as the *pseudo‐robust strategy* as we do not know if it is robust in the sense of Equation (53). For the special distribution of risk aversion where it takes only the two values γ_1_ and γ_2_, it is natural to take $A=(\gamma_{1},\gamma_{2})$. In that case, Equation (54) is easily calculated and compared with the equilibrium strategy. We do this in the next subsection. We leave it to future studies to clarify under which conditions on *A* the value functions in Equations (53) and (54) actually coincide, including whether the set $A=(\gamma_{1},\gamma_{2})$ actually fulfills such conditions. To this end, ideas exploited in Sass and Westphal (2022) for the case of an uncertain drift, have to be properly adapted to the case of a random risk aversion. Moreover, it is encouraging that Balter and Schweizer (2021) find that Equations (53) and (54) coincide in their one‐period model.

Finally, we comment on the areas of time‐dependent risk aversion and horizon‐dependent risk aversion. The case of consumption with time‐varying relative risk aversion has been studied by Steffensen (2011). There, the bold approach from classical state‐dependent utility, corresponding to the approach in Equation (2.1) where the integral is with respect to time, leads to semi‐explicit results. However, the currency unit issues are overlooked and utility of consumption at different points in time with different risk aversions, γ(t), is simply added up. Working with certainty equivalents and the equilibrium approach to time‐inconsistency would also there be a delicate alternative, beyond the scope of our paper, though. In contrast, in the area of horizon‐dependent risk aversion, the problem is time‐inconsistent by construction. There the risk aversion for consumption at time *t*, when standing at time $t_{0},t>t_{0}$, depends on $t-t_{0}$, that is, $\gamma (t-t_{0})$. The appearance of *t*_0_ in the argument makes the problem time‐inconsistent. Again, currency unit issues may arise, and working with certainty equivalents may repair, in that case without necessarily complicating the problem mathematically as it was time‐inconsistent already before introducing certainty equivalents. A recursive approach to the time‐inconsistency of horizon‐dependent risk aversion, without paying attention to the currency unit issues, was presented by Andries et al. (2019).

### 5.2 Calculation of the welfare loss

To quantify the differences between the different approaches, we calculate the welfare loss compared to our approach for power utility.

To do so, we need to explicitly calculate the corresponding equilibrium value functions: from Theorem 3.5, we know that

$$\begin{array}{c} J^{\hat{\pi }}\left(t,x\right)=\int {\left(u^{\gamma }\right)}^{-1}\left(Y^{\gamma }\left(t,x\right)\right)d\Gamma \left(\gamma \right)\;. \end{array}$$

More specifically, for power utility preferences, we obtain with

$$\begin{array}{cc} {\left(u^{\gamma }\right)}^{-1}\left(\cdot \right)={\left(1-\gamma \right)}^{\frac{1}{1-\gamma }}{\left(\cdot \right)}^{\frac{1}{1-\gamma }}\;,\;Y^{\gamma }\left(t,x\right) & =\frac{1}{1-\gamma }{\left(g^{\gamma }\left(t\right)\right)}^{\gamma }x^{1-\gamma }, \end{array}$$

where the functions $g^{\gamma }$ are determined for all γ via the ODE (35) such that

$$\begin{array}{c} J^{\hat{\pi }}\left(t,x\right)=\int {\left(g^{\gamma }\left(t\right)\right)}^{\frac{\gamma }{1-\gamma }}\;x\;\;d\Gamma \left(\gamma \right)\;. \end{array}$$

For the case of two possible risk aversions $\left(\gamma_{1},\gamma_{2}\right)$, realized with probabilities $\left(p\left(\gamma_{1}\right),p\left(\gamma_{2}\right)\right)$, this collapses to

$$\begin{array}{c} J_{\gamma_{1},\gamma_{2}}^{\hat{\pi }}\left(t,x\right)=\left(p\left(\gamma_{1}\right)g^{\gamma_{1}}{\left(t\right)}^{\frac{\gamma_{1}}{1-\gamma_{1}}}+p\left(\gamma_{2}\right)g^{\gamma_{2}}{\left(t\right)}^{\frac{\gamma_{2}}{1-\gamma_{2}}}\right)x, \end{array}$$

where the functions $g^{\gamma_{1}}$ and $g^{\gamma_{2}}$ are characterized by the ODEs (40) and (41).

For the case of only one possible risk aversion γ, we recover the optimal value function of a Merton investor

$$\begin{array}{c} J_{\gamma }^{\hat{\pi }}\left(t,x\right)=g^{\gamma }{\left(t\right)}^{\frac{\gamma }{1-\gamma }}x, \end{array}$$

where the function $g^{\gamma }$ is characterized by the ODE (44).

Translating this in what we refer to as the expected risk aversion investor, the welfare loss of an investor using this strategy compared to the equilibrium investor is determined via solving the following equation for the relative welfare loss β:

(55) $$\begin{array}{c} J_{\gamma_{1},\gamma_{2}}^{\hat{\pi }}\left(0,\left(1-\beta \right)x\right)=J_{E[\gamma ]}^{\hat{\pi }}\left(0,x\right)\;. \end{array}$$

The relative welfare loss β of the pseudo‐robust investor that is facing the two possible risk aversions $\left(\gamma_{1},\gamma_{2}\right)$ compared to the equilibrium investor, is analogously determined via solving the following equation for β:

(56) $$\begin{array}{c} J_{\gamma_{1},\gamma_{2}}^{\hat{\pi }}\left(0,\left(1-\beta \right)x\right)=\min\left\{J_{\gamma_{1}}^{\hat{\pi }}\left(0,x\right),J_{\gamma_{2}}^{\hat{\pi }}\left(0,x\right)\right\}\;. \end{array}$$

The results for β by solving Equations (55) and (56) for the parameters

(r,α,σ,T)=(0.02,0.06,0.2,100),

and risk aversions

$$\gamma_{1}=0.5,\;\gamma_{2}=4,$$

with the possible two scenarios

$$p\left(\gamma_{1}\right)=p\left(\gamma_{2}\right)=0.5\;\text{and}\;p\left(\gamma_{1}\right)=0.8,\;p\left(\gamma_{2}\right)=0.2,$$

are collected in Table 1. It is clearly visible that the investor using the expected risk aversion and the pseudo‐robust investor suffer a wealth loss compared to the equilibrium investor, and the one of the robust investor is larger than the one of the expected risk aversion one. Moreover, the loss is increasing from the 50/50 to the 80/20 case, as for the second case, the more conservative risk aversion γ_2_, which corresponds to a lower Merton proportion, is less likely than in the first case.

**TABLE 1:** Welfare loss for the two possible scenarios 50/50 and 80/20.

β	50/50	80/20
Expected risk aversion	65.56%	85.75%
Pseudo‐robust	76.66%	95.56%

**FIGURE 3:**
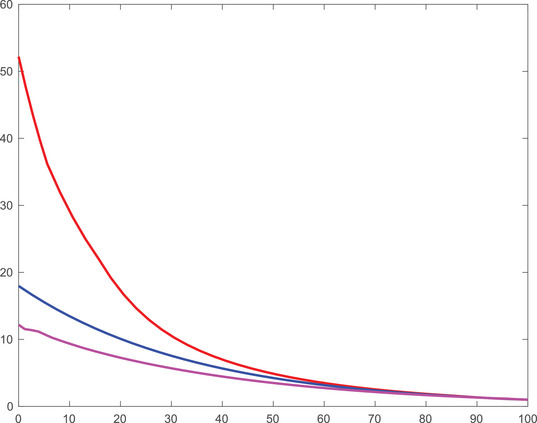
Value functions of the equilibrium investor (red), the expected risk aversion investor (blue) and the pseudo‐robust investor (purple) as a function of time for fixed wealth x=1 in the 50/50 scenario. [Colour figure can be viewed at wileyonlinelibrary.com]

**FIGURE 4:**
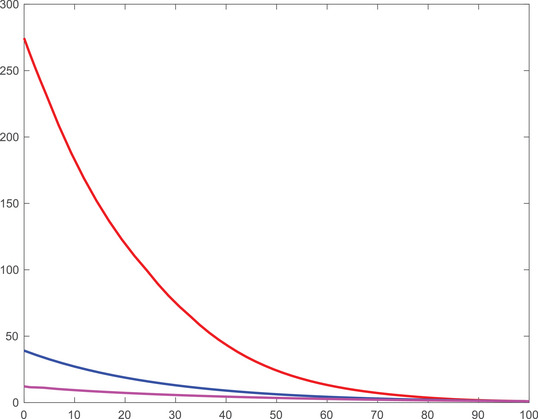
Value functions of the equilibrium investor (red), the expected risk aversion investor (blue), and the pseudo‐robust investor (purple) as a function of time for fixed wealth x=1 in the 80/20 scenario. [Colour figure can be viewed at wileyonlinelibrary.com]

The value functions of the three cases that we discuss are illustrated in Figures 3 and 4, for both the 50/50 and the 80/20 case over time for a normalized wealth of x=1. In line with the welfare loss, the value function of the equilibrium investor dominates the ones of the expected risk aversion investor and the pseudo‐robust investor. Notably, they all converge to a single value towards the investment horizon, confirming the observations made in Figures 1 and 2 on a strategy level.

## Data Availability

Data sharing not applicable to this article as no datasets were generated or analyzed during the current study.

## ENDNOTES

1 And even if that was the case, we could impose the slight restriction that the investment horizon is smaller than the explosion time of the local solution, that is, $T<T_{\infty }$.

2 It is easy to see that ν actually is a density by checking that ∫dν(γ)=1.
